# Iridium Nanoparticle-Gated Janus Nanomachines for Enzyme-Controlled Drug Delivery

**DOI:** 10.1021/acsanm.6c01043

**Published:** 2026-08-03

**Authors:** Beatriz Mayol, Marta González-Jiménez, Alfredo Sánchez, Susana Portela, Sandra Pradana-López, Carolina López-Benet, Teresa Moreno, RuiYuan Cai, Paloma Martínez-Ruiz, Narcisa Martínez-Quiles, Paula Díez, Ramón Martínez-Máñez, Irene Ojeda, Diana Vilela, Reynaldo Villalonga

**Affiliations:** † Nanosensors and Nanomachines Group, Department of Analytical Chemistry, Faculty of Chemistry, 16734 Complutense University of Madrid, Madrid 28040, Spain; ‡ Department of Electrical and Computer Engineering, Rice University, Houston, Texas 77005, United States; § Instituto Interuniversitario de Investigación de Reconocimiento Molecular y Desarrollo Tecnológico (IDM), Universitat Politècnica de València, Universitat de València, Camino de Vera, s/n., València 46022, Spain; ∥ Department of Immunology, Ophthalmology and ENT, School of Medicine, Complutense University, Madrid 28040, Spain; ⊥ Unidad Mixta de Investigación En Nanomedicina y Sensores, Universitat Politècnica de València, Instituto de Investigación Sanitaria La Fe (IIS La Fe), Valencia 46026, Spain; # CIBER de Bioingeniería, Biomateriales y Nanomedicina, Instituto de Salud Carlos III, Bellaterra 08913, Spain; ∇ Unidad Mixta UPV-CIPF de Investigación en Mecanismos de Enfermedades y Nanomedicina, Universitat Politècnica de València, Centro de Investigación Príncipe Felipe, Eduardo Primo Yúfera 3, València 46012, Spain

**Keywords:** nanomachines, nanorobots, drug delivery systems, enzymes, Janus-nanoparticles

## Abstract

Controlled drug delivery systems (DDSs) have emerged as a promising alternative to conventional therapies, aiming to optimize drug release profiles and improve therapeutic outcomes while reducing systemic side effects. In this work, iridium-gated Janus nanomachines are proposed as enzyme-regulated platforms for smart and autonomous drug delivery. These hybrid nanostructures consist of mesoporous silica nanoparticles asymmetrically functionalized with gold nanoparticles bearing surface-anchored enzymes on the metallic hemisphere, while the opposite mesoporous face incorporates pH-responsive molecular gates based on aminophenylboronic acid and d-lactose-functionalized iridium nanoparticles, enabling efficient cargo loading and motility behavior. To evaluate nanomachine operation, two enzymatic systems were employed: glucose oxidase (GOx) and a tandem esterase/alcohol oxidase (AOX) system, using spectrophotometric monitoring of the model cargo release. Both platforms exhibited well-defined release kinetics and good correlation between substrate concentration and high selectivity toward their respective inputs. Motility studies revealed the appearance of two particle populations in the presence of glucose or methanol, consistent with enhanced motion driven by catalytic reactions at the iridium nanoparticle surface upon enzymatic hydrogen peroxide generation. Finally, the GOx-based nanomachine loaded with the antitumoral drug doxorubicin (DOXO) demonstrated efficient cellular internalization, preserved biocompatibility, and enhanced antitumoral activity in HeLa cells. Overall, this work reinforces the potential of enzyme-controlled Janus nanomachines as multifunctional platforms integrating autonomous motion and stimulus-responsive drug delivery for advanced therapeutic applications.

## Graphical abstract

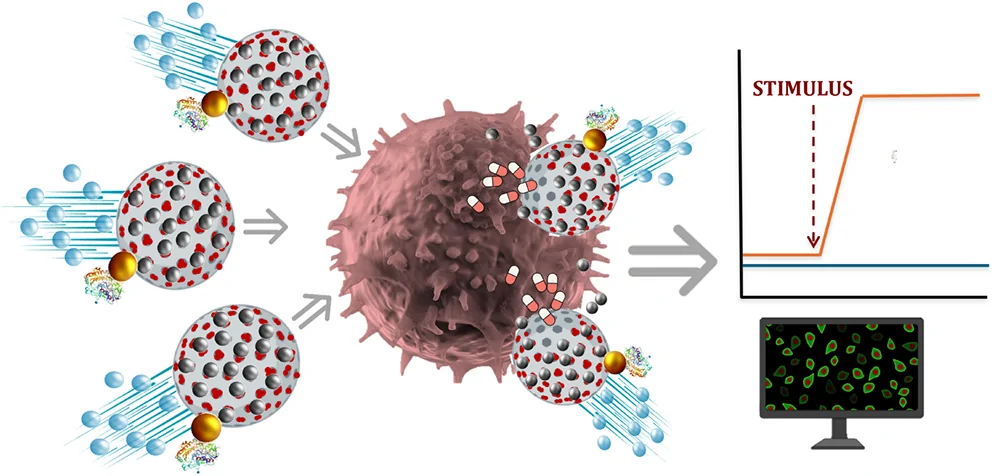

## Introduction

The development of effective therapeutic strategies for a wide range of diseases remains a major challenge in modern medicine. Conventional pharmacological treatments often suffer from variable bioavailability and poorly controlled exposure profiles, resulting in fluctuating drug levels that may reduce therapeutic efficacy and increase the risk of systemic side effects.^1–3^ In this context, the time-controlled delivery of therapeutic agents to diseased tissues to minimize off-target effects has become a central goal in drug design. Advanced drug delivery systems have emerged to address these challenges by enhancing therapeutic precision while minimizing systemic toxicity. Consequently, in the last decades, considerable research efforts have focused on the development of smart delivery platforms that respond to specific stimuli characteristic of pathological microenvironments with broad relevance in biomedicine and diagnostics.^4–8^ Among these approaches, liposomes,^9^ polymeric nanoparticles,^10^ inorganic scaffolds,^11^ and emerging enzymatic nanomotors^12,13^ have attracted significant attention, as they are designed to enhance tissue penetration and precisely modulate therapeutic release. Collectively, these nanomaterials improve drug bioavailability, prolong circulation time, and promote site-specific accumulation, thereby reducing systemic toxicity.^14^ Within this framework, Janus architectures have gained increasing relevance owing to their intrinsic asymmetry, which allows the spatial integration of distinct chemical and biochemical functions within a single nanoparticle through toposelective synthetic strategies.^15,16^ This versatility has facilitated the development of advanced Janus nanomotors as smart delivery systems.^17–20^ In particular, mesoporous silica nanoparticles (MSNs) represent an attractive platform due to their high cargo-loading capacity, biocompatibility, and tunable size and pore structure.^21,22^ Furthermore, MSNs can be functionalized with stimulus-responsive molecular gates that ensure a “zero-release” state until activation by specific physical, chemical, or biochemical signals,^7,23,24^ enabling the design of Janus MSN-based nanomachines with autonomous motion and/or controlled release capabilities.^20,25,26^ Regarding controlled drug release, the incorporation of enzymes as functional control units has enabled the development of increasingly sophisticated nanomachines. In earlier systems, enzymatic catalytic activity has been exploited both as a propulsion source in nanomotors^6^ and as a biochemical trigger,^25,27–29^ converting specific substrates into chemical signals that induce gate opening in mesoporous supports. This dual role provides an effective strategy to couple autonomous motion with stimulus-responsive cargo release within a single nanoplatform.^30^

In this study, we developed a novel hybrid Janus nanoplatform based on Janus Au-MSNs (J-Au) capped with iridium nanoparticle (IrNP)-based gating scaffolds (see Scheme 1). In these systems, one MSN hemisphere is decorated with gold nanoparticles (AuNPs) and functionalized with enzyme-based control units that trigger drug release and regulate the nanomotor response, while the opposite silica face incorporates pH-responsive gating architectures composed of aminophenylboronic acid and d-lactose-functionalized IrNPs, which enable efficient cargo loading, enzyme-controlled release, and self-propulsion behavior. The nanodevice is assembled by selectively modifying the mesoporous face of the Janus colloids (J-Au)^26^ with boronic acid moieties, followed by cargo loading using Ru­(bipy)_3_Cl_2_ as a model probe and doxorubicin (DOXO) as a chemotherapeutic agent. The gate-like ensemble is subsequently constructed through the immobilization of previously prepared d-lactose-modified IrNPs via reversible boronic acid ester bonds. The complete platform is subsequently biofunctionalized with distinct enzymatic systems to obtain nanodevices that exhibit different functional responses. Specifically, glucose oxidase (GOx) is employed for the preparation of J-Au/GOx-bor-(Ru)-Ir and J-Au/GOx-bor-(DOXO)-Ir, whereas the combined action of esterase and alcohol oxidase (AOX) is used for J-Au/AOX/esterase-bor-(Ru)-Ir, with the resulting nanomotor behavior being dictated by the specific enzymatic pathway involved. As shown in Scheme 1, cargo release is triggered when the enzymatic reaction generates acidic products that locally decrease the pH, leading to the cleavage of the boronic ester linkage between d-lactose and aminophenylboronic acid. Conversely, when the enzymatic substrate produces H_2_O_2_, the intrinsic catalytic activity of the IrNPs induces the generation of oxygen, enabling the nanomachine propulsion.

**Scheme 1:**
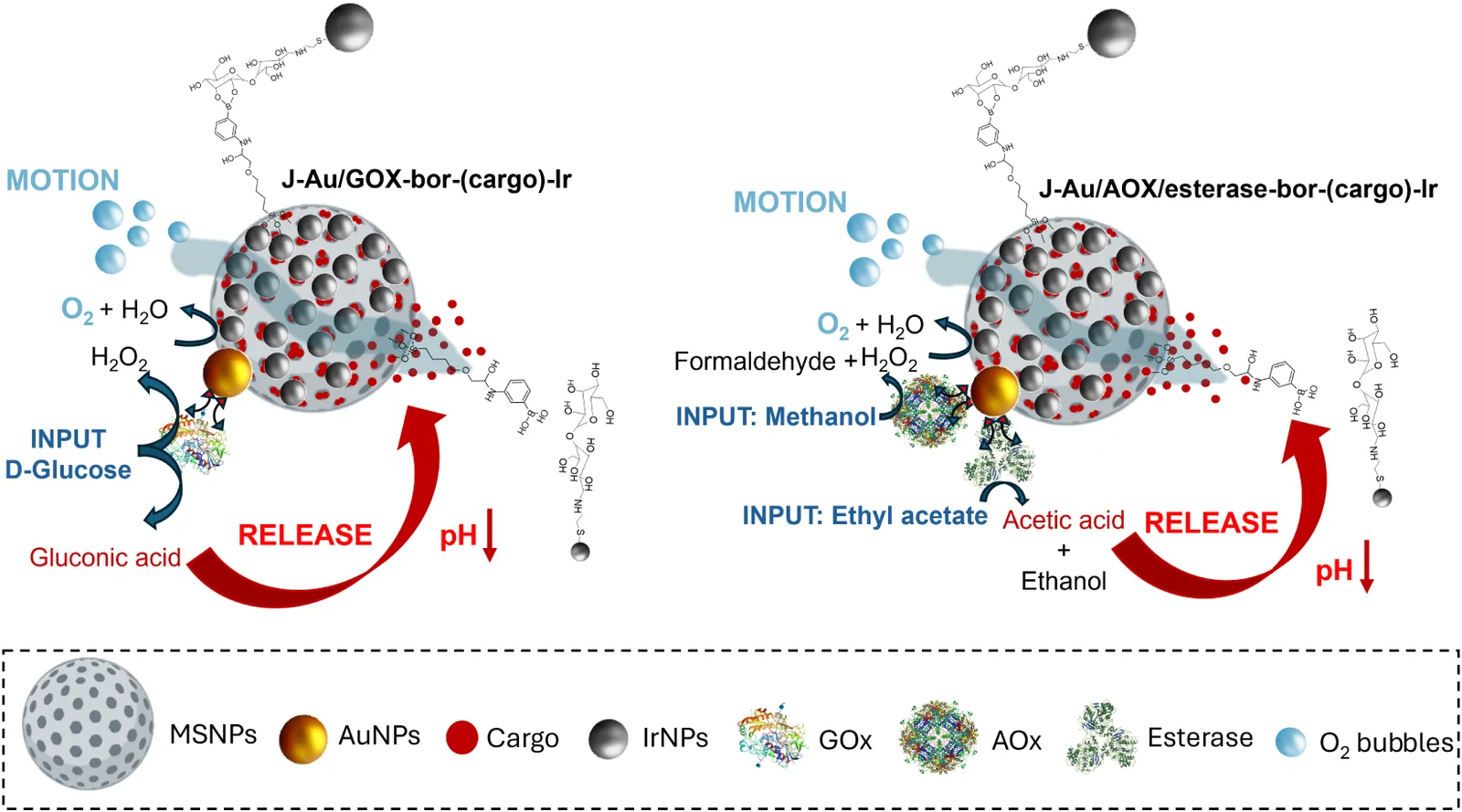
Proposed Mechanism For Enzyme-Activated Operation of Janus Nanomachines Equipped with IrNPs-Based Molecular Gates for Controlled Drug Release^a^

a J-Au/GOx-bor-(Ru)-Ir and J-Au/GOx-bor-(DOXO)-Ir are functionalized with GOx, and J-Au/AOX/esterase-bor-(Ru)-Ir incorporates esterase/AOX.

## Results and Discussion

Scheme 2 depicts the experimental procedure for constructing the enzyme-controlled nanomachines J-Au/GOx-bor-(Ru or DOXO)-Ir and J-Au/AOX/esterase-bor-(Ru)-Ir. First, MCM-41-type mesoporous silica nanoparticles were synthesized, and subsequently, J-Au was prepared via a paraffin wax-based Pickering emulsion, following a methodology previously reported by our group.^26^

**Scheme 2:**
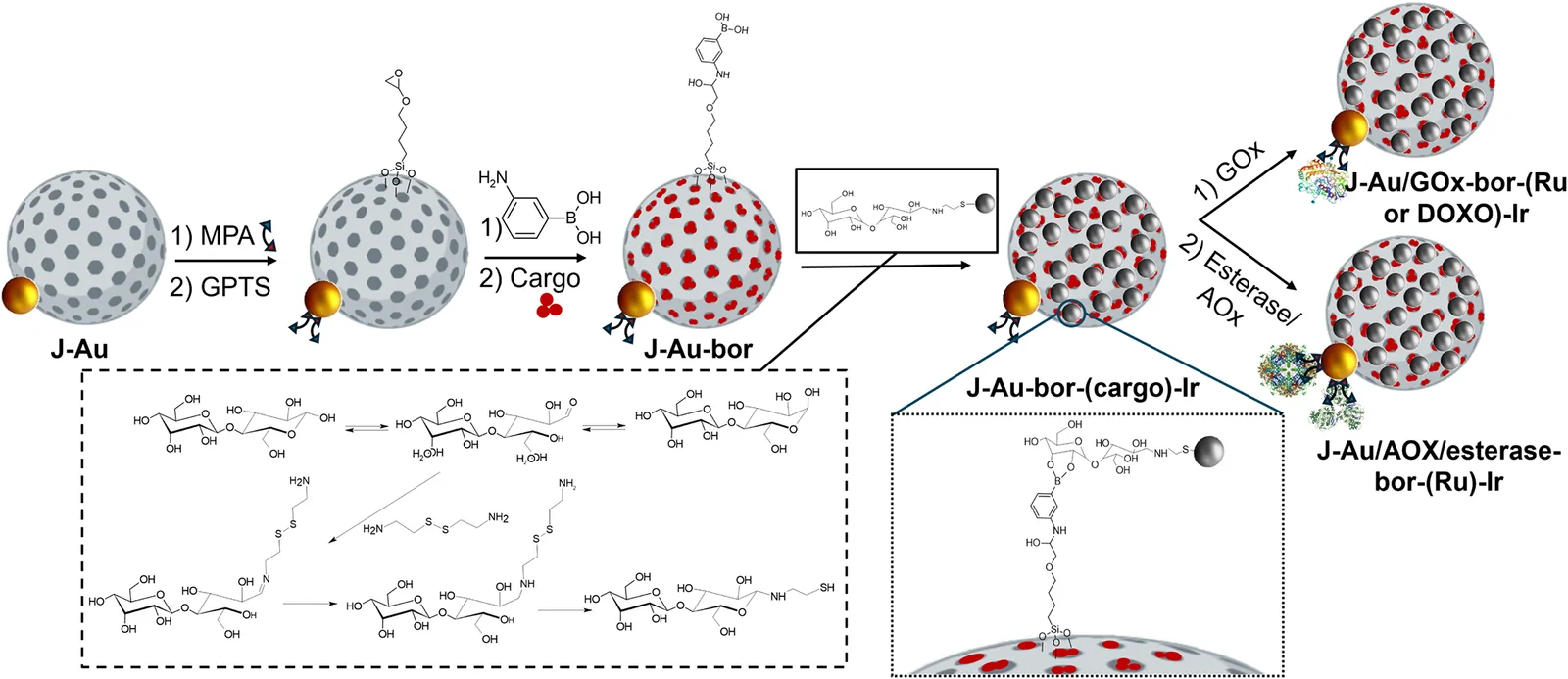
Schematic Preparation of the Nanomachines: J-Au/GOx-bor-(Ru)-Ir GOx Controlled IrNPs-Gated Janus Nanomachines Loaded with Ru­(bipy)_3_Cl_2_; and J-Au/AOX/esterase-bor-(Ru)-Ir Esterase/AOX Controlled IrNPs-Gated Janus Nanomachines Loaded with Ru­(bipy)_3_Cl_2_; J-Au/GOx-bor-(DOXO)-Ir GOx Controlled IrNPs-Gated Janus Nanomachines Loaded with DOXO

To enable enzyme immobilization, the gold face of J-Au was functionalized with 3-mercaptopropionic acid (MPA). Next, the mesoporous silica surface was modified via silane chemistry using 3-(glycidyloxypropyl)­trimethoxysilane (GPTMS), thereby introducing epoxy functionalities. These epoxy groups were subsequently reacted with 3-aminophenylboronic acid through nucleophilic ring opening of the epoxide moiety by the amino group. Unreacted epoxy groups were finally blocked by treatment with an excess of ethylenediamine. To obtain the intermediate solid J-Au-bor-(cargo)-Ir, the cargo molecules were first loaded into the mesoporous silica pores, followed by the assembly of the gating mechanisms using previously prepared d-lactose-functionalized IrNPs. The gate was anchored through the formation of reversible boronic ester linkages, thereby effectively sealing the cargo within the pores. Subsequently, the appropriate enzyme, either GOx or the esterase/AOX pair, was covalently anchored onto the carboxylic acid groups presented on the gold surface via EDC/NHS coupling chemistry, yielding the functional nanomachines J-Au/GOx-bor-(Ru or DOXO)-Ir or J-Au/AOX/esterase-bor-(Ru)-Ir, respectively. The resulting nanodevices, J-Au/GOx-bor-(Ru)-Ir and J-Au/AOX/esterase-bor-(Ru)-Ir, were loaded with a colorimetric and fluorescent ruthenium complex, whereas J-Au/GOx-bor-(DOXO)-Ir was loaded with the antitumoral agent DOXO for subsequent evaluation as a therapeutic system *in vitro* using HeLa cells.

The nanomaterials were characterized by using different techniques. Transmission electron microscopy (TEM) revealed the characteristic spherical morphology of MSNs, with an average diameter of 106 ± 5 nm (*n* = 50, confidence interval) and confirmed the presence of the ordered hexagonal pore arrangement typical of MCM-41 (Figure 1A). TEM images of IrNPs revealed a mean particle size of 2.0 ± 0.1 nm (*n* = 50, confidence interval) (Figure S1, Supporting Information). For the hybrid J-Au-bor-(cargo)-Ir nanostructures, an average diameter of 113 ± 12 nm (*n* = 20, confidence interval) was observed, along with the presence of multiple IrNPs homogeneously distributed over the silica surface (Figure 1B), supporting their function as molecular gate components.

**Figure 1:**
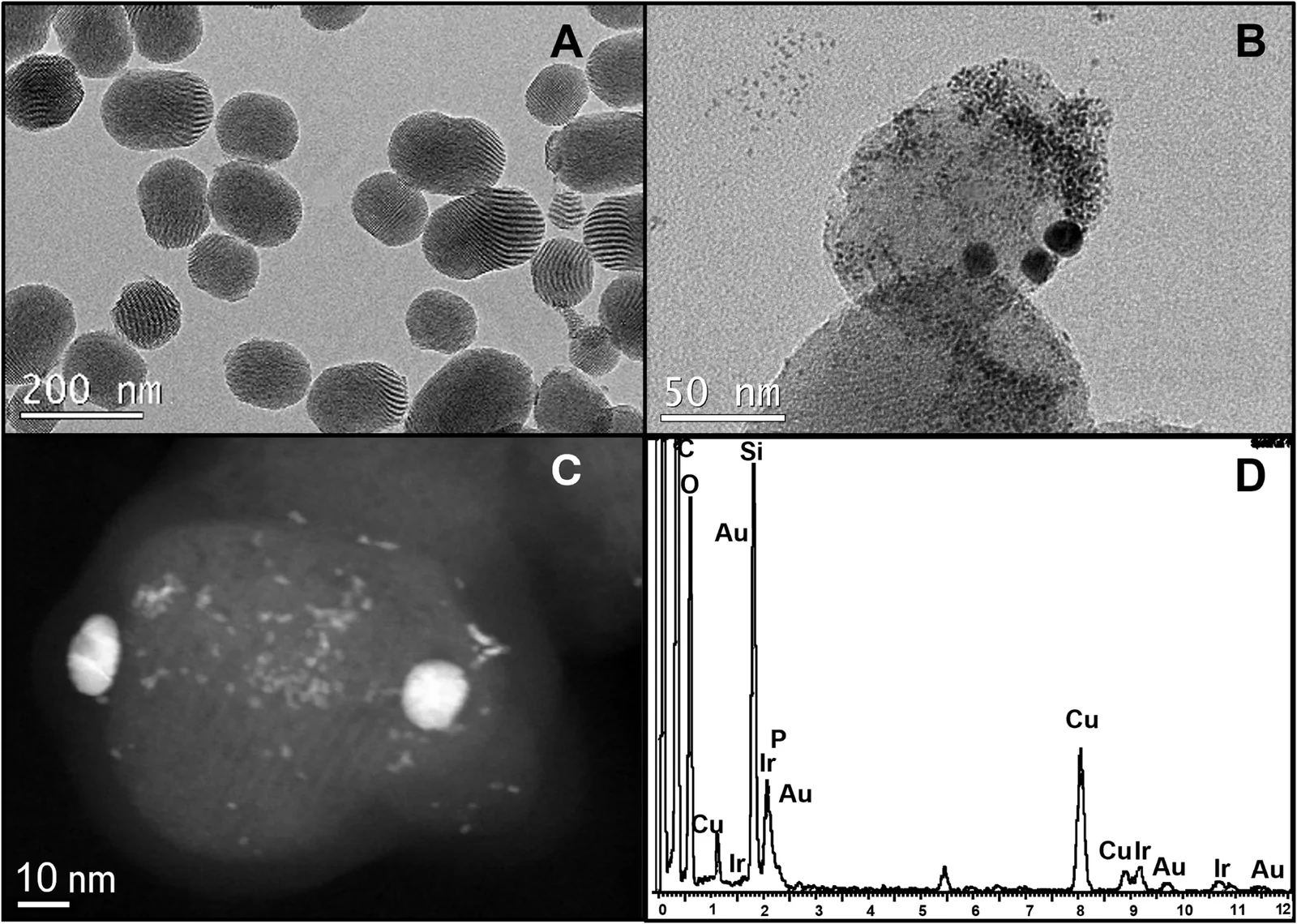
Characterization of solid J-Au-bor-(cargo)-Ir: TEM images of (A) MCM-41 and (B) solid J-Au-bor-(cargo)-Ir, (C) STEM images, and (D) EDX of J-Au-bor-(cargo)-Ir.

Further characterization of the Janus-type nanomaterial was performed by scanning transmission electron microscopy (STEM) coupled with energy-dispersive X-ray spectroscopy (EDX) (Figure 1C and 1D). The STEM image showed the hybrid architecture of the J-Au-bor-(cargo)-Ir nanostructure (Figure 1C), while the EDX analyses confirmed the elemental composition of the system, revealing distinct signals corresponding to oxygen, silicon, gold, and iridium (Figure 1D).

Additional structural characterizations of solid J-Au-bor-(Ru)-Ir were carried out by powder X-ray diffraction (XRD), thermogravimetric analysis (TGA), and Fourier transform infrared (FT-IR) spectroscopy (see Figure S2). The low-angle XRD pattern shown in Figure S2A confirms that J-Au-bor-(Ru)-Ir retains the characteristic mesostructural order of MCM-41 materials. The diffractograms exhibit the typical reflections of a hexagonally ordered mesoporous framework, with well-defined Bragg peaks indexed to the (100), (110), and (200) planes, indicating the preservation of the cylindrical pore architecture associated with MCM-41.^31^ In the high diffraction angle region, the XRD patterns of J-Au and J-Au-bor-(Ru)-Ir displayed additional diffraction peaks corresponding to metallic Au and Ir NPs, which can be indexed to the (111), (200), (220), and (311) crystallographic planes, in agreement with previous reports.^32,33^ These signals provide clear evidence of the successful incorporation of gold and iridium NPs onto the mesoporous silica scaffold.

To monitor the synthetic sequence, thermal analyses were performed (Figure S2C). All nanomaterials exhibited a minor mass loss between 60 and 90 °C, which is attributed to the desorption of physisorbed water. For pristine MSNs, a gradual additional mass loss up to 1000 °C was observed, consistent with the progressive condensation and decomposition of surface silanol groups. In contrast, the J-Au and Janus boronic acid-functionalized MSNs (J-Au-bor) materials displayed a second, more pronounced degradation step, which can be ascribed to the thermal decomposition of silane moieties and thiol-based organic linkers responsible for anchoring the AuNPs to the silica surface, as well as to the organic components of the molecular gate. Solid J-Au-bor-(Ru)-Ir exhibited a significantly higher mass loss up to 900 °C, which is attributed to the thermal decomposition of the ruthenium complex within mesoporous channels, further supporting successful cargo loading. Figure S2D displays the FT-IR spectra of these nanomaterials, exhibiting the characteristic vibrational bands of mesoporous silica MCM-41 type. In all samples, a broad and strong band between 3750 and 3100 cm^–1^ was observed, corresponding to the O–H stretching vibration of adsorbed water molecules and hydrogen-bonded surface silanol groups in the MCM-41. The peak at 1680 cm^–1^ is the bending vibration of adsorbed water molecules. Bands at 1090, 800, and 469 cm^–1^ correspond to asymmetric stretching, symmetric stretching, and bending vibration of Si–O–Si bonds, respectively. The band centered at 550 cm^–1^ is ascribed to a tetrahedral bending of the Si–O bond.^34^ In the J-Au, J-Au-bor, and particularly in J-Au-bor-(Ru)-Ir, additional bands appear in the 2800–3000 cm^–1^ region, which are assigned to the symmetric and asymmetric stretching modes of aliphatic C–H groups, confirming successful organic functionalization of the silica surface.^35^ Finally, weak adsorption bands around 1500 cm^–1^ observed in J-Au-bor-(Ru)-Ir indicate the presence of the encapsulated ruthenium complex.^36^

Figure S3 in the Supporting Information presents the nitrogen adsorption–desorption isotherms and corresponding pore size distributions of MCM-41, J-Au, and J-Au-bor-(Ru)-Ir using the BET and BJH models. As shown in Figure S3A, the pristine MSN and J-Au samples exhibit type IV isotherms, characteristic of mesoporous materials, with specific surface areas of 1090 and 837 m^2^ g^–1^, respectively. In contrast, sample J-Au-bor-(Ru)-Ir displays a type III isotherm, indicative of nonmesoporous behavior, along with a markedly reduced surface area of 410 m^2^ g^–1^. This transition can be attributed to the incorporation of the ruthenium complex and the presence of the molecular gate, which partially obstructs the mesoporous channels. BJH pore size analysis further supports this interpretation (Figure S3B, Supporting Information), revealing an average pore diameter of approximately 2.2 nm for the initial materials and a slight decrease in pore volume upon incorporation of AuNPs. However, in the case of J-Au-bor-(Ru)-Ir, the extensive pore blockage prevents the determination of a well-defined pore size distribution.

The total cargo loading capacity of J-Au-bor-(Ru)-Ir was quantified by alkaline hydrolysis followed by spectrophotometric analysis, which revealed a dye loading of 12.4 mg g^–1^ J-Au-bor-(Ru)-Ir by weight. To evaluate whether the molecular gate assembly was able to release the encapsulated cargo under acidic conditions, a spectrophotometric release assay was carried out at 454 nm using J-Au-bor-(Ru)-Ir in the presence of 200 mmol L^–1^ HCl. As shown in Figure S4 in the Supporting Information, acidification of the medium immediately triggered the release of the dye confined within the silica pores. In contrast, no detectable release was observed under neutral conditions, demonstrating that the designed molecular gates operate effectively and open selectively in response to an acidic stimulus.

After establishing the structural features and gating behavior of the iridium-gated Janus nanomachines J-Au-bor-(Ru)-Ir, this platform was subsequently employed as a modular scaffold for the construction of enzyme-responsive nanodevices with distinct enzymic configurations and different sensing and regulatory elements. Specifically, enzyme-controlled system J-Au/GOx-bor-(Ru)-Ir was prepared through the immobilization of GOx. In parallel, J-Au/AOX/esterase-bor-(Ru)-Ir was engineered by covalently immobilizing esterase and AOX onto the gold surface while loading [Ru­(bpy)_3_]­Cl_2_ within the MSN framework as the luminescent probe (see Scheme 1).

In parallel, J-Au/AOX/esterase-bor-(Ru)-Ir was engineered by covalently immobilizing esterase and AOX onto the gold nanoparticle while incorporating [Ru­(bpy)_3_]­Cl_2_ as the luminescent probe.

In the hybrid GOx-controlled iridium-gated Janus nanomachine J-Au/GOx-bor-(Ru)-Ir, GOx catalyzes the oxidation of d-glucose, producing gluconic acid and hydrogen peroxide (H_2_O_2_). The gluconic acid generated induces a local decrease in pH, which cleaves the boronic acid ester-based gate-like ensemble located at the mesoporous face. This cleavage disrupts the linkage between the IrNPs-based molecular gate and the silica surface mediated by d-lactose, thereby triggering the release of the encapsulated cargo, as illustrated in Figure 2A. Simultaneously, H_2_O_2_ produced during the reaction undergoes catalytic decomposition on the IrNP surface, generating O_2_ bubbles that enhance diffusion and promote the propulsion of the J-Au/GOx-bor-(Ru)-Ir nanomachines. In addition, the response of J-Au/GOx-bor-(Ru)-Ir to substrate concentration was evaluated. Figure 2B shows a clear dose-dependent relationship between d-glucose concentration and the amount of dye released. These results demonstrate that enzymatic activation controls the concentration-dependent opening of the molecular gate, thereby regulating the overall extent of cargo release. The enzymatic activity of GOx was subsequently quantified to assess the catalytic loading of J-Au/GOx-bor-(Ru)-Ir, revealing a specific activity of 0.63 U mg^–1^ with respect to J-Au/GOx-bor-(Ru)-Ir.

**Figure 2:**
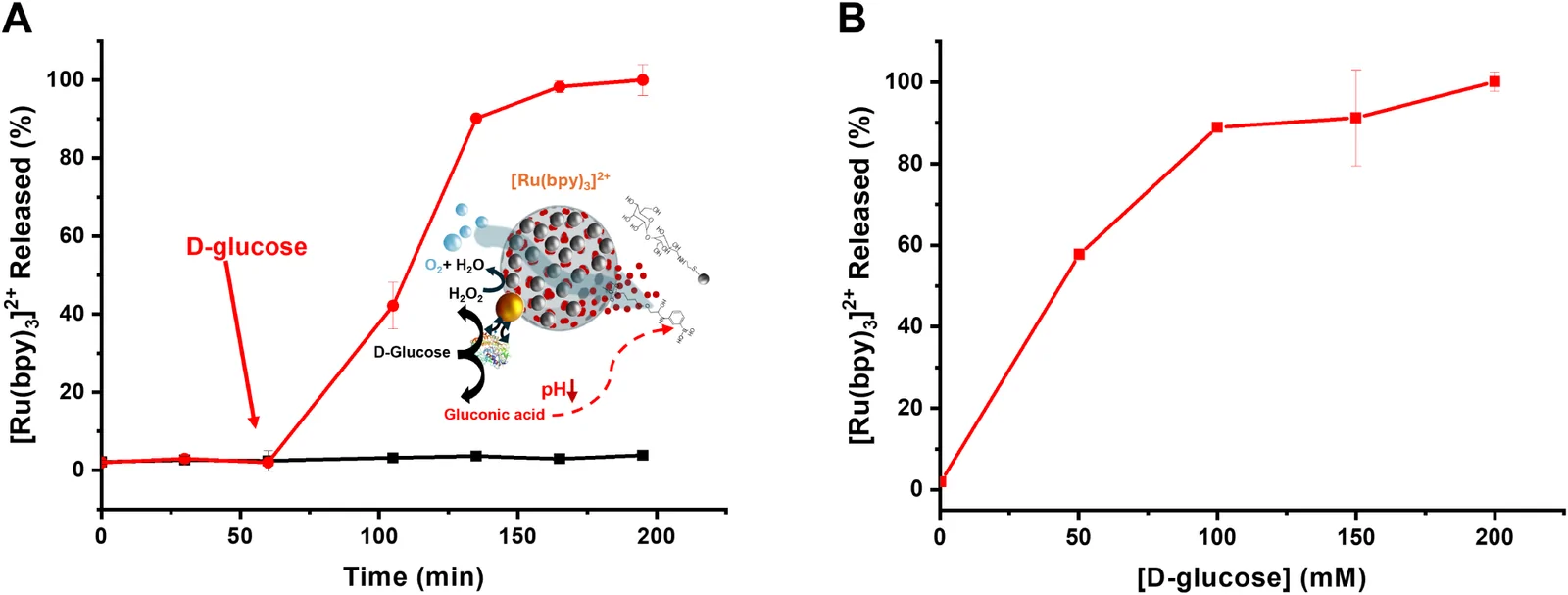
(A) Kinetics release profile and (B) effect of substrate concentration on the release activity after 2 h of incubation of J-Au/GOx-bor-(Ru)-Ir. Conditions: 5 mmol L^–1^ PB (pH 7.5).

In contrast to J-Au/GOx-bor-(Ru)-Ir, the hybrid J-Au/AOX/esterase-bor-(Ru)-Ir system operates through a bienzymatic module immobilized on the gold domain. In this configuration, esterase recognizes ester-containing substrates and generates the corresponding carboxylate. The resulting local acidification disrupts the linkage between the IrNP-based molecular gate and the silica surface, thereby enabling the release of the encapsulated dye. In parallel, AOX catalyzes the oxidation of alcohols to produce H_2_O_2_, which acts as the chemical fuel required to drive nanomachine motion (Scheme 1).

Figure 3A–C shows the release kinetics of [Ru­(bpy)_3_]­Cl_2_ from J-Au/AOX/esterase-bor-(Ru)-Ir in the absence and presence of the specific substrates for each enzyme, as well as in the presence of both substrates simultaneously. Figure 3A corresponds to experiments performed with ethyl acetate, Figure 3B with methanol, and Figure 3C with a combination of both, along with a substrate-free control. For J-Au/AOX/esterase-bor-(Ru)-Ir, the highest cargo release was observed in the presence of ethyl acetate, followed by the combination of both substrates, whereas no significant release occurred when methanol was used alone. This trend is attributed to the fact that esterase activity is required to generate the acidic trigger necessary for gate opening, while methanol does not induce acid formation. Moreover, methanol may partially inhibit esterase activity, as alcohols are reaction products of esterase-catalyzed hydrolysis. Consequently, elevated methanol concentrations can interfere with efficient substrate turnover (Figure 3D). In all cases, negligible dye release was detected in the absence of ethyl acetate, confirming the strict enzymatic dependence of the gating mechanism. Figure 3D shows the response of J-Au/AOX/esterase-bor-(Ru)-Ir to increasing ethyl acetate concentrations in the presence or absence of 50 mmol L^–1^ MeOH. Although MeOH partially inhibited dye release, a clear dose-dependent response was observed in all systems. This confirms that cargo release is regulated by enzymatic activation of the molecular gate. The enzymatic activities of the bienzymatic J-Au/AOX/esterase-bor-(Ru)-Ir system were also quantified, revealing specific activities of 0.037 U mg^–1^ and 0.69 U mg^–1^ for AOX and esterase, respectively.

**Figure 3:**
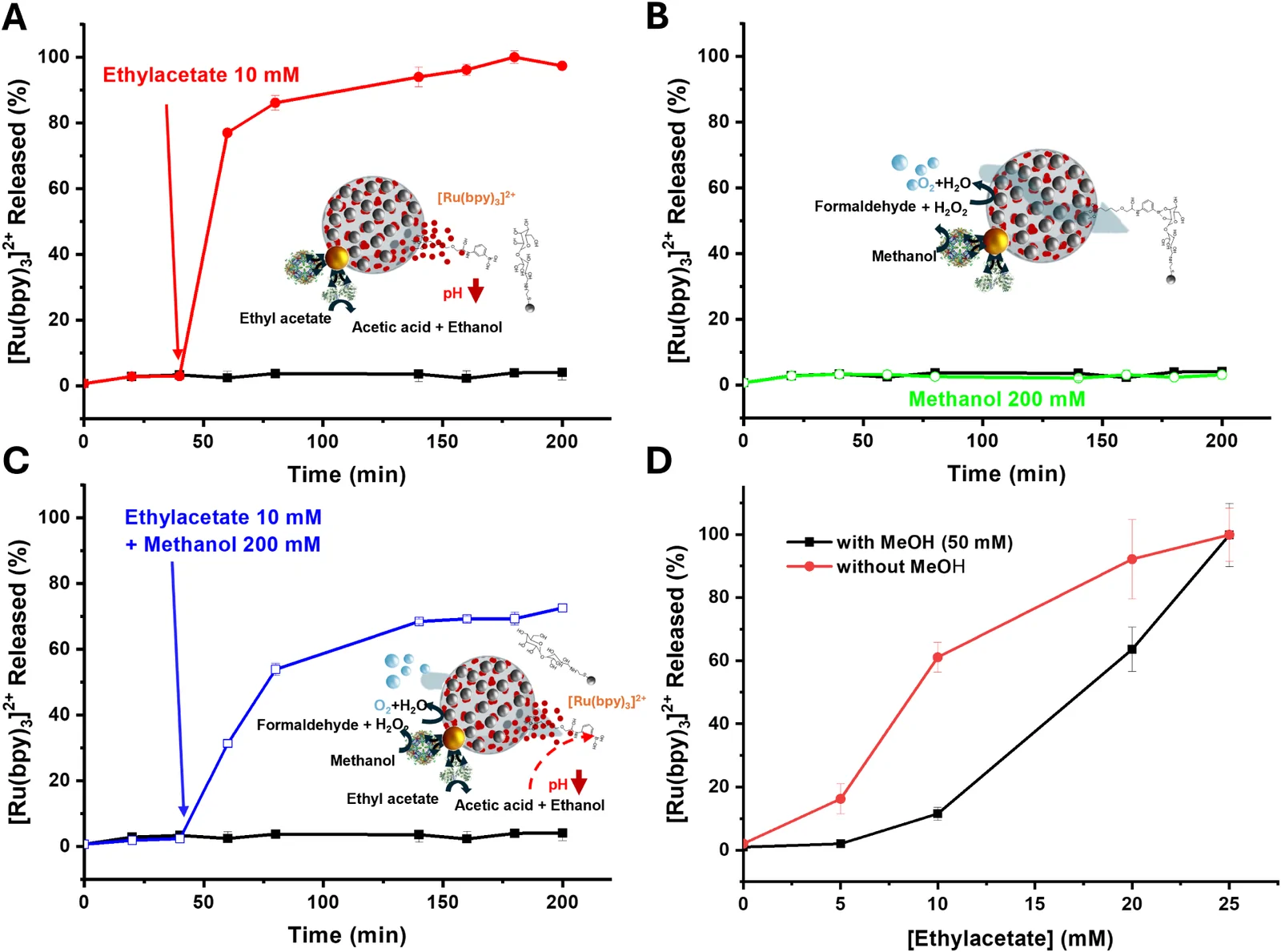
Kinetics release profile in response to (A) ethyl acetate, (B) methanol, and (C) both ethyl acetate and methanol for J-Au/AOX/esterase-bor-(Ru)-Ir and (D) effect of the release of ethyl acetate concentration in the presence and absence of MeOH, respectively. Conditions: 5 mmol L^–1^ PB (pH 7.5).

To further verify that cargo release is strictly dependent on enzymatic activity, the catalytic modules immobilized on each nanomachine (GOx in J-Au/GOx-bor-(Ru)-Ir and esterase/AOX in J-Au/AOX/esterase-bor-(Ru)-Ir) were thermally inactivated by boiling for 10 min prior to testing. Release experiments were then repeated in the presence of the corresponding substrate. As shown in Figure S5 (Supporting Information), nanomachines containing inactivated enzymes exhibited negligible dye release, demonstrating that the molecular gate remains closed in the absence of active biocatalysis and confirming the essential role of the enzymatic trigger in the operating mechanism.

Figure 4 depicts the selectivity evaluation against molecules that are chemically related to the enzymatic substrates employed in J-Au/GOx-bor-(Ru)-Ir and J-Au/AOX/esterase-bor-(Ru)-Ir. The J-Au/GOx-bor-(Ru)-Ir nanomachine exhibited negligible dye release upon its incubation with a panel of sugars such as d-fructose, d-galactose, and sucrose. In contrast, exposure to d-glucose induced a pronounced release response, confirming its role as the specific activating stimulus and consistent with the elevated d-glucose levels characteristic of the tumor microenvironment (Figure 4A). A complementary specificity study was performed for J-Au/AOX/esterase-bor-(Ru)-Ir to identify potential molecular interferents. The bienzyme-based nanomachine was evaluated with a diverse set of esters, alcohols, and sugars, including ethyl butyrate, ethyl bromoacetate, d-glucose, sucrose, ethanol, and 1-propanol. Figure 4B shows significant cargo release exclusively for ester-containing substrates, in agreement with the catalytic profile of esterase, which selectively hydrolyzes ester bonds. No appreciable release was detected for the remaining molecules, confirming the high selectivity of J-Au/AOX/esterase-bor-(Ru)-Ir and its strict dependence on ester-based activation.

**Figure 4:**
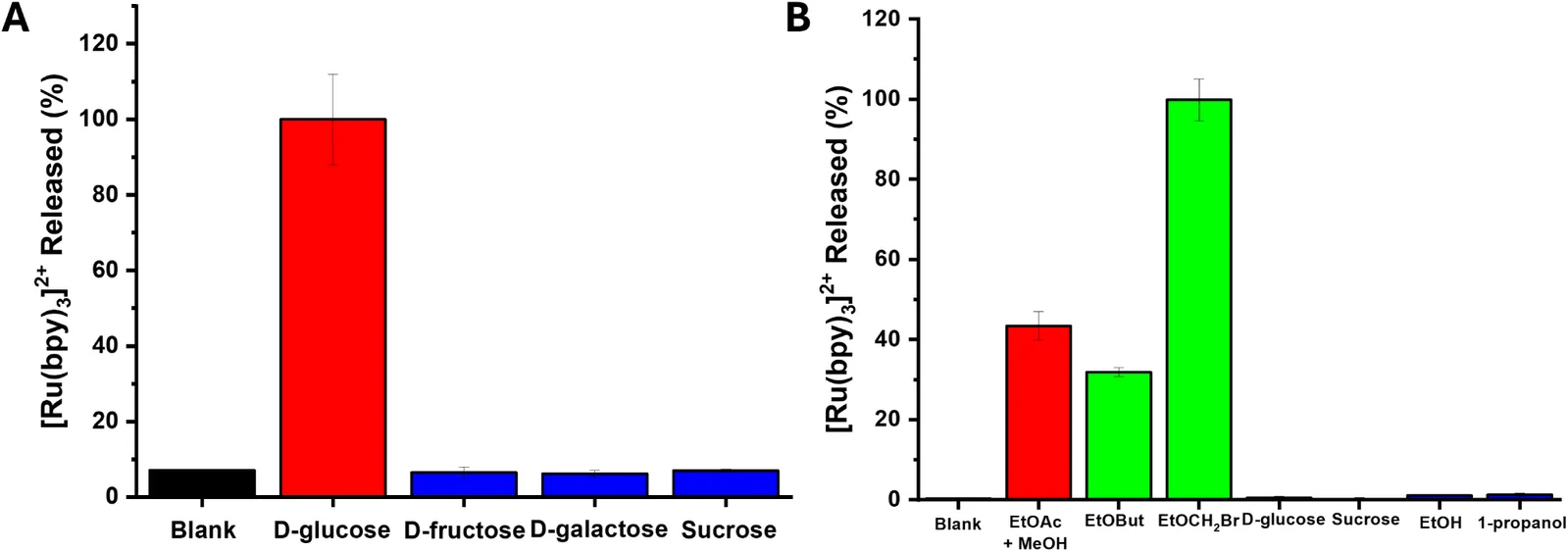
Release percentage of (A) J-Au/GOx-bor-(Ru)-Ir in the presence of different sugars and (B) J-Au/AOX/esterase-bor-(Ru)-Ir in the presence of different esters, sugars, and alcohols. Control experiments without analytes. Conditions: 5 mmol L^–1^ PB (pH 7.5), and measurements were driven after 2 h of incubation.

As illustrated in Scheme 1, the iridium-based molecular gates of nanomachines J-Au/GOx-bor-(Ru)-Ir and J-Au/AOX/esterase-bor-(Ru)-Ir are capable of generating O_2_ in the presence of hydrogen peroxide, which is one of the enzymatic products of GOx and AOX, respectively. On this basis and in order to demonstrate the motile behavior of J-Au/GOx-bor-(Ru)-Ir and J-Au/AOX/esterase-bor-(Ru)-Ir, their diffusion coefficients were evaluated by nanoparticle tracking analysis (NTA) using a NanoSight NS300 system. The resulting trajectories were subsequently processed and analyzed with custom-developed R scripts. *x*–*y* trajectories of 20 individual nanoparticles (100–400 nm) were recorded for 30 s in 5 mM PBS containing increasing glucose concentrations (0–100 mM). Mean square displacement (MSD) values were calculated from the trajectories to determine the diffusion coefficients of passive nanoparticles (*D*_0_) and glucose-powered nanomotors (*D*_eff_). As expected, J-Au/GOx-bor-(Ru)-Ir passive nanomachines exhibited Brownian motion, whereas the presence of glucose promoted enhanced diffusion, with *D*_eff_ increasing in a glucose-concentration-dependent manner (Figure 5A–C). As expected, in the case of J-Au/AOX/esterase-bor-(Ru)-Ir (Figure 5D–F), no enhancement in particle diffusion was observed upon the addition of ethyl acetate, since its hydrolysis does not generate H_2_O_2_ and therefore cannot trigger catalytic propulsion. In contrast, the addition of methanol resulted in enhanced particle diffusion and longer trajectories. This behavior is attributed to the oxidation of methanol by AOX, as depicted in Scheme 1, which produces H_2_O_2_ that is subsequently decomposed at the IrNP surface, driving the nanomotor propulsion. Nevertheless, the increase in diffusion was markedly lower than that observed for the GOx-functionalized nanomotors. This reduced propulsion can be ascribed to both the lower catalytic activity of AOX compared with that of GOx and the lower amount of AOX immobilized on the nanomotors, as AOX shares the available surface with esterase in the bienzymatic cascade.

**Figure 5:**
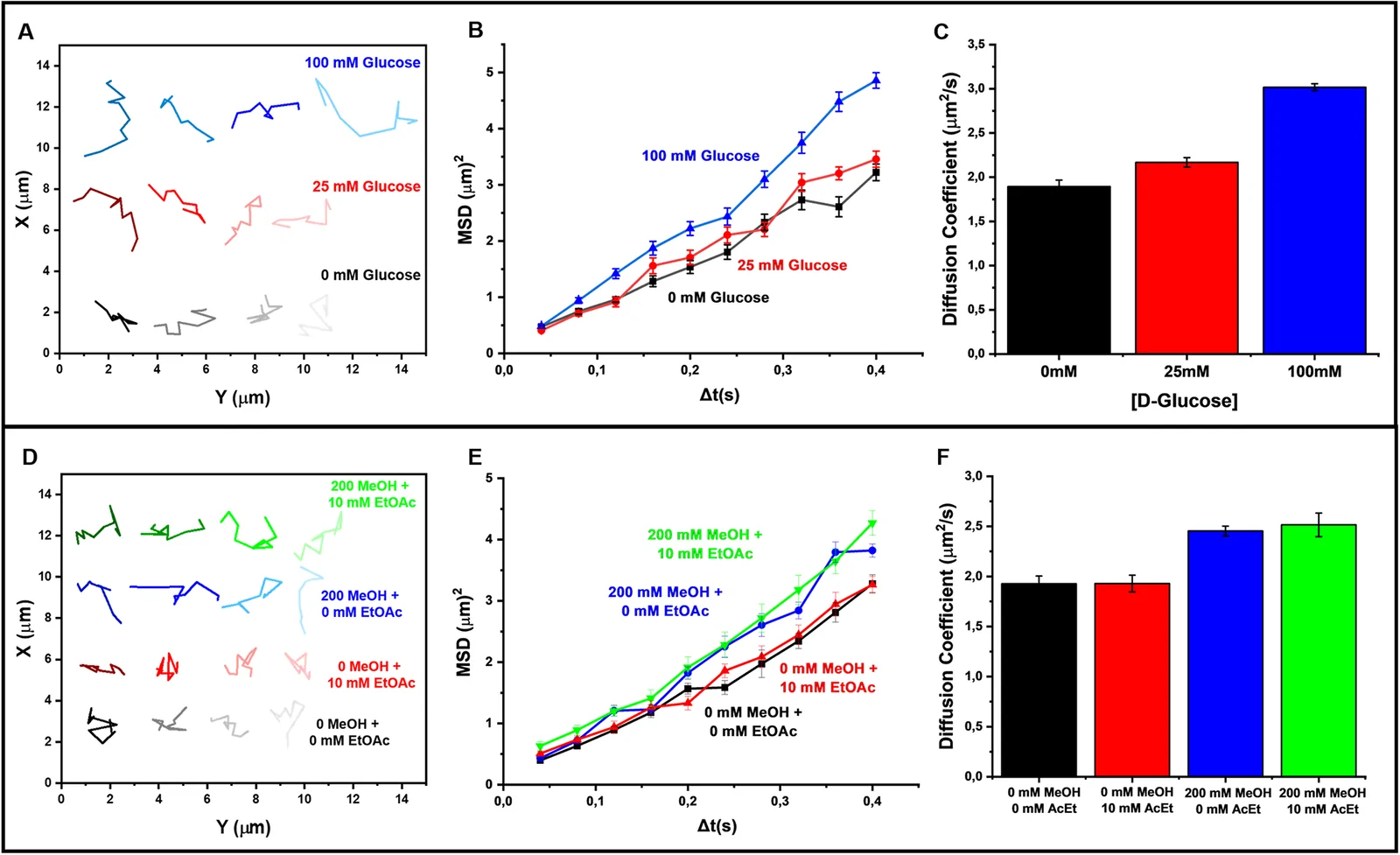
Diffusion evaluation of J-Au/GOx-bor-(Ru)-Ir (A–C) and J-Au/AOX/esterase-bor-(Ru)-Ir (D-F) in 5 mM PBS, pH 7.5. (A) Representative trajectories of J-Au/GOx-bor-(Ru)-Ir nanoparticles for 25 s. (B) Mean-squared displacements (MSDs) of J-Au/GOx-bor-(Ru)-Ir nanoparticles. (C) Diffusion coefficients calculated from the MSDs at different d-glucose concentrations of 0, 25, and 100 mM d-glucose concentration (*N* = 20). (D) Representative trajectories of J-Au/AOX/esterase-bor-(Ru)-Ir nanoparticles for 25 s. (E) Mean-squared displacements (MSDs) of J-Au/AOX/esterase-bor-(Ru)-Ir nanoparticles. (F) Diffusion coefficients calculated from the MSDs at different MeOH and ethyl acetate concentrations (*N* = 20).

Once the proper operation of the nanomotor had been established, its potential as an enzyme-controlled drug-delivery system was evaluated. To this end, the J-Au/GOx-bor-(Ru)-Ir model was selected as a platform for *in vitro* on-demand drug delivery using HeLa cancer cells as a cellular model, as this nanomachine requires high d-glucose concentrations to operate. Notably, this requirement makes the system particularly suitable for tumor-related applications, since the tumor microenvironment is intrinsically characterized by elevated d-glucose levels that can locally activate the nanodevice. For this purpose, an analogue of J-Au/GOx-bor-(Ru)-Ir was prepared from J-Au-bor-(Ru)-Ir by incorporating the chemotherapeutic agent DOXO in place of the Ru complex, yielding the drug-loaded nanomachine J-Au/GOx-bor-(DOXO)-Ir.

First, the release kinetics of DOXO from J-Au/GOx-bor-(DOXO)-Ir were investigated by monitoring the absorbance at 480 nm in the absence and presence of d-glucose as the INPUT stimulus. As shown in Figure 6A, only negligible drug release was observed under control conditions, confirming that J-Au/GOx-bor-(DOXO)-Ir remains closed and efficiently retains DOXO. In contrast, the addition of d-glucose induced a gradual and time-dependent release of DOXO. Spectrophotometric quantification revealed that J-Au/GOx-bor-(DOXO)-Ir released 54.5 μg mL^–1^ after 1400 min of incubation with the stimulus, corresponding to 10.9% of the total DOXO loading and an encapsulation efficiency of 1.1%. These results prove that J-Au/GOx-bor-(DOXO)-Ir is capable of stimulus-responsive, on-demand cargo delivery, operating through the same activation mechanism previously established for J-Au/GOx-bor-(Ru)-Ir.

**Figure 6:**
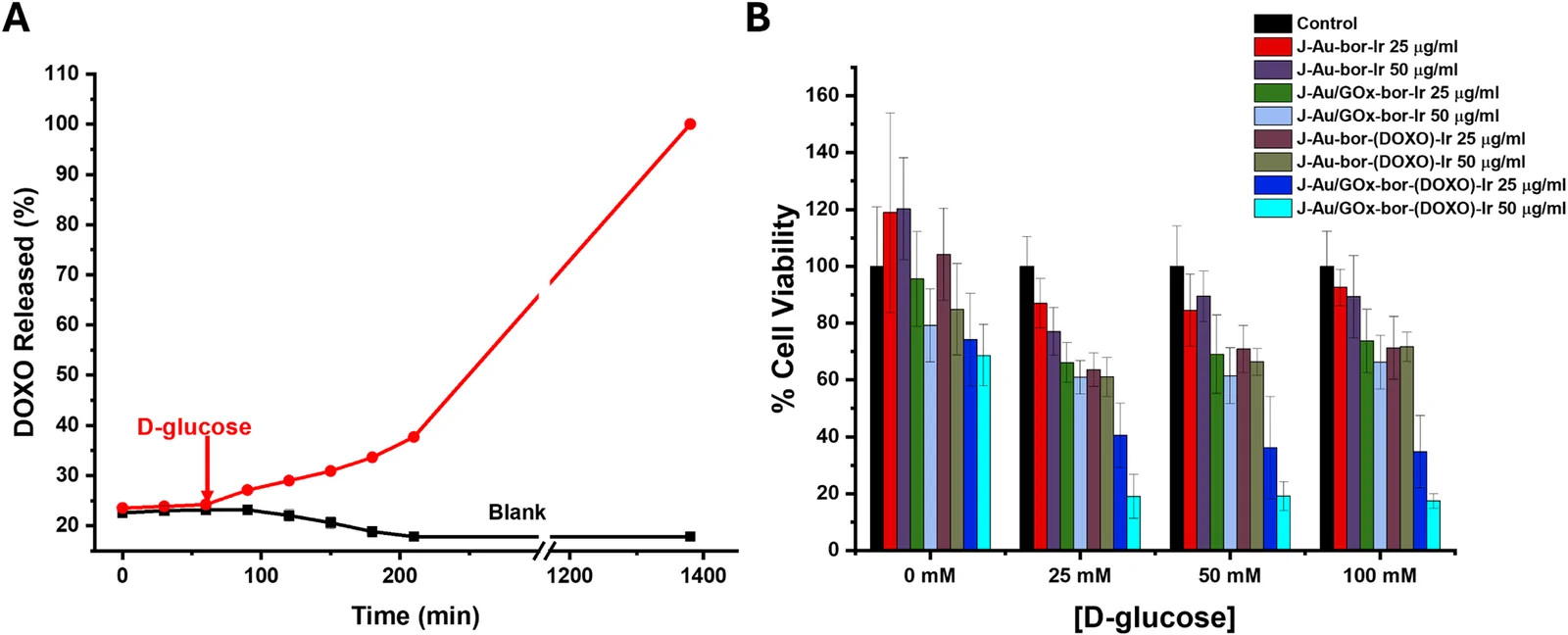
Kinetics release profile in response of J-Au/GOx-bor-(DOXO)-Ir to (A) the absence (black) and presence (red) of 100 mmol L^–1^ d-glucose in 5 mmol L^–1^ PB, pH 7.5 (*n* = 3). (B) Cell viability assays in HeLa cells treated with J-Au/GOx-bor-(DOXO)-Ir and with different controls (J-Au-bor-Ir without anchored GOx and without DOXO, J-Au/GOx-bor-Ir with anchored GOx and without DOXO, and J-Au-bor-(DOXO)-Ir without anchored enzyme and loaded with DOXO) at 25 and 50 μg mL^–1^ final concentrations for 24 h in different d-glucose DMEM media.

For *in vitro* evaluation of J-Au/GOx-bor-(DOXO)-Ir, preliminary cell viability assays were conducted in HeLa cells using a colorimetric MTT-based assay.^37^ Figure 6B exhibits cells exposed to the different controls J-Au-bor-Ir, J-Au/GOx-bor-Ir, and J-Au-bor-(DOXO)-Ir, resulting in viability levels comparable to those of the untreated group, which indicates negligible intrinsic toxicity of the nanocarrier. Nevertheless, when higher concentrations of J-Au/GOx-bor-(DOXO)-Ir, from 25 to 50 μg mL^–1^, were tested, including formulations lacking the enzyme, no statistically significant differences in the reduction of cell viability were observed. This modest decrease is attributed to minimal basal toxicity associated with the inorganic matrix itself.^38^

In the absence of d-glucose, J-Au/GOx-bor-(DOXO)-Ir behaved similarly to the control formulations J-Au-bor-Ir, J-Au/GOx-bor-Ir, and J-Au-bor-(DOXO)-Ir, demonstrating that the drug remains securely confined within the nanocarrier and that no passive leakage of the payload occurs prior to activation of the triggering mechanism. In contrast, when J-Au/GOx-bor-(DOXO)-Ir was administered under glucose-rich conditions, a further decrease in cell viability was observed. This effect suggests that the metabolic stimulus promotes both drug release and enhanced cellular interaction, likely facilitated by the self-propelled motion of the nanomachine. Taken together, these results support the functionality of J-Au/GOx-bor-(DOXO)-Ir as a smart, stimulus-responsive drug delivery platform capable of selective activation in glucose-enriched, tumor-like microenvironments.

Confocal laser scanning microscopy was further employed to investigate the cellular internalization of the prepared nanomachines J-Au/GOx-bor-(DOXO)-Ir and to evaluate the enzyme-controlled release of DOXO, which is selectively activated upon d-glucose. Figure 7 shows representative confocal images in which cell nuclei were stained with Hoechst (blue), and the plasma membrane was labeled with WGA-Alexa Fluor 488 (green). The red fluorescence corresponds to encapsulated DOXO and is observed in HeLa cells treated with J-Au/GOx-bor-(DOXO)-Ir. Untreated cells imaged under identical conditions were used as the controls.

**Figure 7:**
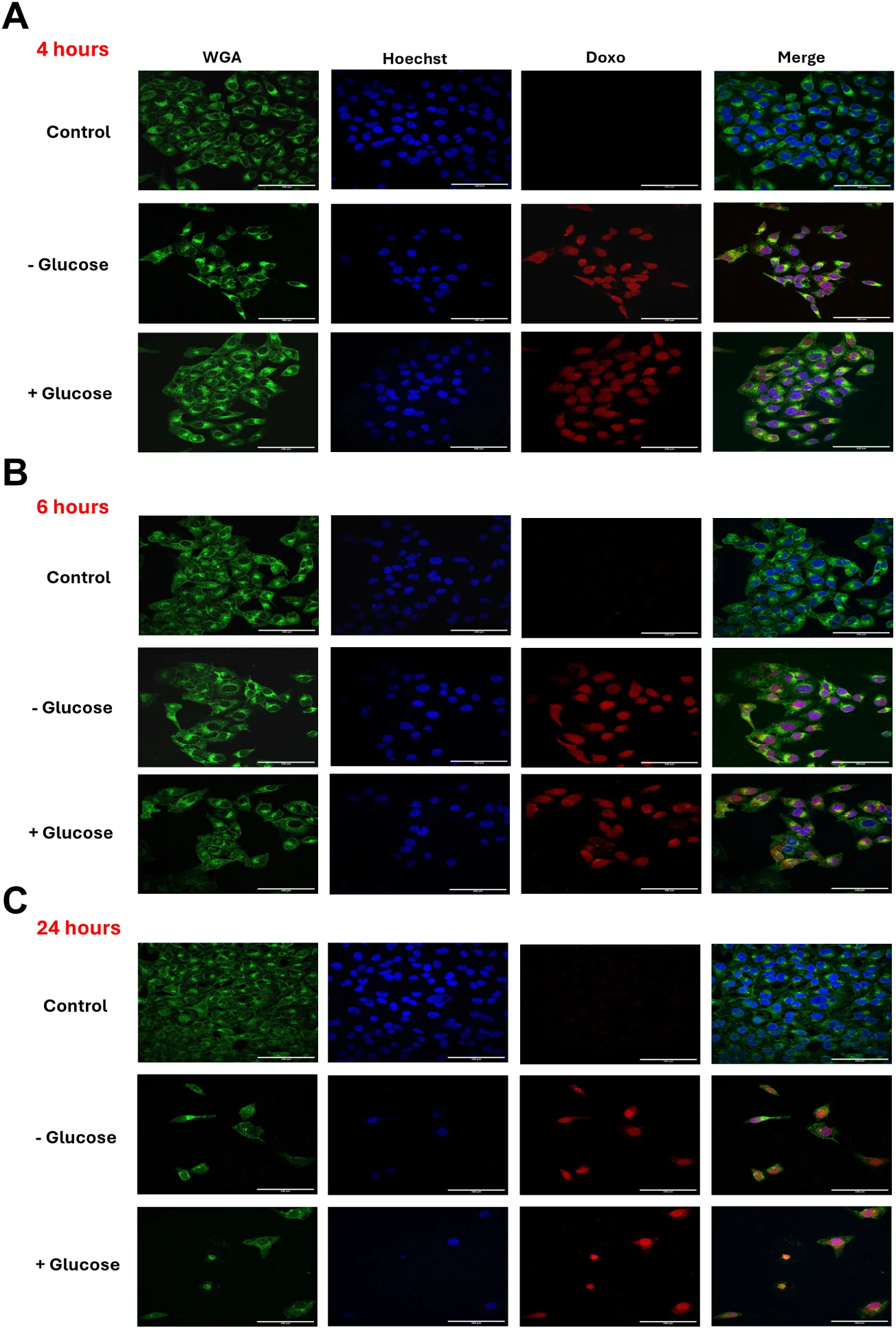
Confocal microscopy images of HeLa cells untreated (control) and incubated with 50 μg mL^–1^ J-Au/GOx-bor-(DOXO)-Ir in the presence of 0 and 25 mmol L^–1^ d-glucose. Images acquired after 4 h (A), 6 h (B), and 24 h (C), showing Alexa Fluor-488 conjugated WGA, Hoechst, DOXO, and combined (merge) staining. Scale bar: 100 μm.

To assess the influence of incubation time and d-glucose availability, HeLa cells were incubated with J-Au/GOx-bor-(DOXO)-Ir at 50 μg mL^–1^ for 4, 6, and 24 h in culture media containing either 0 or 25 mmol L^–1^ d-glucose. Confocal images confirm that J-Au/GOx-bor-(DOXO)-Ir is efficiently internalized by HeLa cells under all tested conditions. At early incubation times (4 and 6 h), cell morphology remained largely preserved, independently of d-glucose concentration. However, after 24 h in glucose-rich medium with activated J-Au/GOx-bor-(DOXO)-Ir nanomachines, a pronounced reduction in cell density together with evident morphological alterations was observed. These changes are consistent with drug release and enhanced cytotoxicity relative to the corresponding controls. Interestingly, a noticeable decrease in cell density was also detected in cells treated with J-Au/GOx-bor-(DOXO)-Ir in glucose-free medium. This effect is likely attributable to the absence of an essential carbon source, which compromises cellular homeostasis and viability independently of drug release.

Overall, these observations indicate that intracellular DOXO release from J-Au/GOx-bor-(DOXO)-Ir is preferentially promoted under glucose-rich conditions through the proposed enzyme-mediated activation mechanism, thereby reinforcing the potential of these nanomachines as a stimulus-responsive therapeutic platform.

## Conclusions

This study demonstrates the versatility and potential of enzyme-regulated IrNP-gated Janus nanomachines as advanced multifunctional platforms for controlled drug delivery. A central feature of these systems is the incorporation of iridium nanoparticle-based molecular gates, which act not only as sealing elements but also as catalytically active components of nanodevices. By integrating asymmetric chemical functionalities, stimulus-responsive molecular gates, and catalytic enzymatic units, these nanostructures have exhibited precise spatiotemporal control over cargo release while enabling autonomously enhanced diffusion. The modular design allows selective activation in response to specific biochemical INPUTs, highlighting the suitability of these systems for applications in complex biological environments. Overall, enzyme-controlled Janus nanomachines represent a promising approach for the development of next-generation smart therapeutics, providing a foundation for future exploration of metabolically responsive, site-selective drug-delivery strategies.

## Materials and Methods

### Preparation of Iridium Nanoparticles (IrNPs) Functionalized with d-Lactose

Iridium nanoparticles were synthesized according to an established protocol with slight modifications.^39^ Briefly, 25 mg of sodium borohydride (NaBH_4_) and 525 mg of CTAB were dissolved in 90 mL of Milli-Q water. In parallel, 38.8 mg of hydrated iridium­(III) chloride was dissolved in 50 mL of Milli-Q water to generate the metal precursor solution. This solution was subsequently added dropwise to the reducing mixture and subjected to ultrasonic irradiation for 10 min to promote homogeneous nucleation. A rapid color transition from yellow (Ir^3+^) to deep black (Ir^0^) was observed, indicative of the formation of metallic Ir NPs. The resulting colloidal dispersion was characterized using a TEM microscope and stored at room temperature until further use.

To functionalize the surface of the prepared IrNPs, a thiolated lactose derivative was synthesized *in situ* and directly conjugated to the iridium nanoparticles. Lactose (584.28 mM) was dissolved in 10 mL of Milli-Q water, followed by the addition of 15.2 mg of cysteamine dihydrochloride. The reaction mixture was stirred at room temperature for 2 h to allow the Schiff base formation. Subsequently, 150 mg of sodium cyanoborohydride was added to selectively reduce the imine intermediates, and the reaction was allowed to proceed for 15 h. Residual imine groups were further reduced by the addition of 38.8 mg of NaBH_4_, and the mixture was stirred for an additional 1 h at room temperature to ensure a complete conversion to secondary amines. The functionalization step was completed by adding 140 mL of the as-prepared IrNP dispersion to the reaction mixture, followed by overnight stirring at room temperature to promote thiol-mediated surface anchoring. Upon completion, the reaction volume was concentrated to approximately 20 mL by controlled evaporation. The resulting product was purified by repeated washing with methanol to remove excess CTAB, unbound lactose derivatives, and residual reducing agents.

### Preparation of Dye-Loaded IrNPs-Gated Janus-Au MSNs (J-Au-bor-(cargo)-Ir)

Selective surface functionalization of the J-Au was carried out through a sequential modification strategy. To functionalize the gold-exposed face, 50 μL of 3-mercaptopropionic acid (MPA) was added to a suspension of J-Au nanoparticles (50 mg) in 5 mL of methanol. The mixture was stirred at room temperature for 1 h to promote thiol-gold chemisorption. The nanoparticles were then isolated by centrifugation, thoroughly washed with methanol to remove excess ligand, and redispersed in 5 mL of a 45 mM solution of (3-glycidyloxypropyl)­trimethoxysilane (GPTMS) in methanol to selectively functionalize the silica surface. The suspension was stirred for 3 h at room temperature, followed by washing with methanol to remove unreacted silane. Subsequently, the epoxide-functionalized nanoparticles were incubated in 5 mL of a 45 mM solution of 3-aminophenylboronic acid in methanol under continuous stirring at room temperature for 18 h, enabling nucleophilic ring-opening of the epoxide groups. Excess boronic acid was removed by repeated washing with methanol. Remaining epoxide functionalities were then passivated by treatment with a 1% v/v ethanolamine solution in methanol for 1 h at room temperature. After purification by washing with methanol, the resulting functionalized nanoparticles were dried at 60 °C and stored until further use.

Cargo loading was performed by dispersing 50 mg of the functionalized nanoparticles in 15 mL of anhydrous acetonitrile, followed by the addition of 30 mg of [Ru­(bpy)_3_]­Cl_2_. The mixture was transferred to a round-bottom flask equipped with a Dean–Stark apparatus and heated under reflux at 110 °C to enable azeotropic removal of residual water adsorbed within the mesoporous framework. After dehydration, the suspension was stirred for an additional 24 h at room temperature to facilitate dye diffusion and pore loading. The solid was recovered by centrifugation, and residual acetonitrile was removed by heating at 60 °C.

Finally, 50 mg of the dye-loaded nanoparticles was dispersed in 3 mL of sodium phosphate buffer (5 mM, pH 7.5). A spatula-tip amount of free dye and 2 mL of lactose-functionalized iridium nanoparticle dispersion (also prepared in 5 mM phosphate buffer, pH 7.5) were added, and the mixture was gently stirred at room temperature for 15 h to allow supramolecular assembly and gate formation. The resulting nanoparticles were thoroughly washed with the same buffer to remove excess dye and nonanchored IrNPs, yielding the IrNPs-gated Janus nanodevices J-Au-bor-(cargo)-Ir.

### Preparation of Enzyme-Controlled IrNPs-Gated Janus-Au MSNs (J-Au/GOx-bor-(Ru)-Ir and J-Au/AOX/esterase-bor-(Ru)-Ir)

Dye-loaded nanoparticles equipped with molecular gating systems (J-Au/GOx-bor-(Ru)-Ir and J-Au/AOX/esterase-bor-(Ru)-Ir) were employed as a versatile platform for the construction of two different enzyme-responsive nanomachines, differentiated by the nature of the enzymatic effectors: glucose oxidase (GOx), yielding J-Au/GOx-bor-(Ru)-Ir, and a bienzymatic combination of alcohol oxidase (AOX) and esterase, affording J-Au/AOX/esterase-bor-(Ru)-Ir.

To initiate enzyme conjugation, 50 mg of J-Au/AOX/esterase-bor-(Ru)-Ir was dispersed in 5 mL of phosphate buffer (5 mM, pH 7.5). Carboxylic acid groups exposed on the gold face of J-Au-bor-(Ru)-Ir were activated by the addition of 25 mg of *N*-(3-dimethylaminopropyl)-*N*′-ethyl carbodiimide (EDC) and 25 mg of *N*-hydroxysuccinimide (NHS), both dissolved in 1 mL of the same buffer. The suspension was incubated for 30 min at 4 °C under gentle stirring to promote the formation of reactive NHS esters.

The activated nanoparticles were thoroughly washed with 5 mM PB (pH 7.5) to remove residual reagents. Functionalization was then carried out by adding 10 mg of GOx to obtain J-Au/GOx-bor-(Ru)-Ir or 15 mg of the enzyme mixture (AOX + esterase) to produce J-Au/AOX/esterase-bor-(Ru)-Ir. The reaction mixture was incubated for 15 h at 4 °C under stirring, followed by multiple washes with PB to eliminate unbound enzymes.

Following activation, the nanoparticles were thoroughly washed with phosphate buffer (5 mM, pH 7.5) to remove excess coupling reagents. Enzyme immobilization was subsequently carried out by adding 10 mg of GOx to obtain J-Au/GOx-bor-(Ru)-Ir or a mixture of 7.5 mg AOX and 7.5 mg esterase to generate J-Au/AOX/esterase-bor-(Ru)-Ir. The reaction mixtures were incubated for 15 h at 4 °C under continuous stirring to facilitate amide bond formation. Finally, the resulting enzyme-functionalized nanodevices were purified by repeated washing with phosphate buffer to remove unbound enzymes.

### Preparation of DOXO-Loaded GOx-Controlled IrNPs-Gated Janus-Au MSNs (J-Au/GOx-bor-(DOXO)-Ir)

The nanomachine J-Au/GOx-bor-(DOXO)-Ir was prepared through a sequential loading and assembly strategy. Briefly, 20 mg of the functionalized J-Au were dispersed in 1 mL of phosphate buffer (0.1 mol L^–1^, pH 7.5) containing 12 mg of DOXO. The suspension was gently stirred overnight at 4 °C to promote drug diffusion and encapsulation within the mesoporous framework. Subsequently, lactose-functionalized IrNPs, previously dispersed in the same buffer (0.1 mol L^–1^, pH 7.5), were added to the mixture, and the suspension was further stirred overnight at 4 °C to enable gate assembly and pore sealing. The resulting nanodevices were thoroughly washed with phosphate buffer to remove nonencapsulated drug and excess IrNPs and stored at 4 °C until use at a final concentration of 10 mg mL^–1^.

For enzyme conjugation, 10 mg of the obtained solid was dispersed in 1 mL of phosphate buffer (5 mM, pH 7.5). Surface carboxylic groups were activated by the addition of 5 mg of EDC and 5 mg of NHS, both dissolved in the same buffer. The suspension was incubated for 30 min at 4 °C under gentle stirring and subsequently washed with phosphate buffer (0.1 M, pH 7.5) to remove excess coupling reagents. It was then resuspended in 1 mL of phosphate buffer (0.1 M, pH 7.5), and 2 mg of GOx was added. The mixture was stirred for 15 h at 4 °C to allow covalent enzyme immobilization. Finally, the enzyme-functionalized IrNPs-gated Janus nanosystems were purified by three successive washing steps with phosphate buffer and redispersed in 1 mL of buffer, yielding a final concentration of 10 mg mL^–1^ of J-Au/GOx-bor-(DOXO)-Ir.

Control samples were prepared for cell viability assays, omitting either the enzyme, the drug, or both components (J-Au-bor-Ir, J-Au/GOx-bor-Ir, and J-Au-bor-(DOXO)-Ir). All control formulations were adjusted to a final concentration of 10 mg mL^–1^.

The loading capacity of J-Au/GOx-bor-(DOXO)-Ir was evaluated by quantifying the amount of encapsulated DOXO using UV–vis absorption spectroscopy. Specifically, the absorbance of the DOXO solution at 480 nm was measured before and after incubation with the nanoparticles, using a molar absorption coefficient of 13500 L mol^–1^ cm^–1^ at 480 nm. The drug loading content and encapsulation efficiency were calculated according to the following equations:

$$\mathrm{DOXO loading content}\;\left(\%\right)=\frac{m_{\mathrm{DOXO}}}{m_{J‐\mathrm{Au}/\mathrm{GOx}‐\mathrm{bor}‐\left(\mathrm{DOXO}\right)‐\mathrm{Ir}}}\times 100$$

$$\mathrm{Encapsulation efficiency}\;\left(\%\right)=\frac{m_{\mathrm{DOXO}\left(J‐\mathrm{Au}/\mathrm{GOx}‐\mathrm{bor}‐\left(\mathrm{DOXO}\right)‐\mathrm{Ir}\right)}}{m_{DOXO\left(0\right)}}\times 100$$

where *m*_DOXO(J‑Au/GOx‑bor‑(DOXO)‑Ir)_= weight of DOXO in J-Au/GOx-bor-(DOXO)-Ir,

*m*_J‑Au/GOx‑bor‑(DOXO)‑Ir_ = weight of J-Au/GOx-bor-(DOXO)-Ir,

*m*_DOXO(0)_ = initial weight of DOXO in solution

### Release Assays of Nanomachines J-Au/GOx-bor-(Ru)-Ir and J-Au/AOX/esterase-bor-(Ru)-Ir in the Presence of the Respective Substrates

Release assays were performed by dispersing 1.25 mg of nanomachines in 125 μL of phosphate buffer (5 mM, pH 7.5), yielding a final concentration of 10 mg mL^–1^ per assay tube. For each experimental condition, three independent samples and three corresponding blanks were prepared. As an initial control, release experiments were conducted in the presence of hydrochloric acid (200 mM) to validate the pH responsiveness of the molecular gating system for J-Au/GOx-bor-(Ru)-Ir. Subsequently, stimulus-responsive release studies were carried out using the appropriate enzyme substrate, d-glucose for J-Au/GOx-bor-(Ru)-Ir or ethyl acetate, methanol, or their binary mixture for J-Au/AOX/esterase-bor-(Ru)-Ir. The influence of analyte concentration on nanodevice activation was systematically evaluated by recording the absorbance of the released cargo as a function of the analyte concentration added to the system. The release profiles of the encapsulated cargo were monitored by UV–vis spectroscopy, with absorbance measurements recorded at 454 nm at 30 min intervals until stabilization of the release signal was observed.

### Release Assays of Nanomachines J-Au/GOx-bor-(DOXO)-Ir in the Presence of d-Glucose

Release experiments involving J-Au/GOx-bor-(DOXO)-Ir nanomachines were conducted by following the same protocol described for J-Au/GOx-bor-(Ru)-Ir. The stimulus-triggered release of encapsulated drug DOXO was monitored spectrophotometrically at 480 nm. For activated release assays, d-glucose was added at a final concentration of 100 mM (*n* = 3), and absorbance measurements were recorded at 30 min intervals after sample centrifugation. Control experiments were performed in parallel using J-Au/GOx-bor-(DOXO)-Ir dispersions in the absence of d-glucose to assess potential baseline drug leakage under nonstimulated conditions.

### Selectivity Evaluation of J-Au/GOx-bor-(Ru)-Ir and J-Au/AOX/esterase-bor-(Ru)-Ir

The selectivity of the enzyme-responsive J-Au/GOx-bor-(Ru)-Ir and J-Au/AOX/esterase-bor-(Ru)-Ir was evaluated. To this end, 1.3 mg of J-Au/GOx-bor-(Ru)-Ir or J-Au/AOX/esterase-bor-(Ru)-Ir was dispersed in 125 μL of phosphate buffer (5 mM, pH 7.5), respectively. Absorbance measurements were recorded every 30 min during an initial incubation period to confirm the absence of spontaneous cargo release. Subsequently, 25 μL of the corresponding analyte solution were added to each sample, while 25 μL of buffer were added to the blank controls. For J-Au/GOx-bor-(Ru)-Ir, selectivity was assessed using d-glucose, d-fructose, d-galactose, and sucrose (all at 150 mM). For J-Au/AOX/esterase-bor-(Ru)-Ir, the analytes tested included ethyl acetate (10 mM) in the presence and absence of methanol (200 mM), ethyl butyrate (10 mM), ethyl bromoacetate (10 mM), d-glucose (150 mM), sucrose (150 mM), ethanol (50 mM), and 1-propanol (50 mM). After incubation for 120 min at room temperature, samples were centrifuged and the absorbance of the supernatant was measured at 454 nm.

### Nanomachine Diffusion Study of J-Au/GOx-bor-(Ru)-Ir and J-Au/AOX/esterase-bor-(Ru)-Ir

The dynamic behavior of the nanomotors was evaluated by analyzing their *x*–*y* coordinates using the NTA 3.0 software integrated into the Nanosight NS300 system. For each experimental condition, 20 individual nanomotors were selected, previously dispersed at 120 μg/mL in phosphate buffer (5 mM, pH 7.5). The suspensions were prepared in the presence of the corresponding fuels: d-glucose (0, 25, and 100 mM) for J-Au/GOx-bor-(Ru)-Ir nanomachines containing GOx, or MeOH (0 and 200 mM) combined with ethyl acetate (0 or 10 mM) for J-Au/AOX/esterase-bor-(Ru)-Ir nanomachines containing AOX and esterase.

To minimize drift problems associated with fuel addition, the d-glucose or MeOH/AcOEt solutions were preloaded into the instrumental chamber. Subsequently, 800 μL of each suspension was introduced using a syringe, maintaining the temperature at 25 °C inside the Nanosight system. For each concentration, five videos of 30 s were recorded with a frame rate of 30 frames/s.

The *x*–*y* coordinates of each nanoparticle were extracted, assuming two-dimensional motion, and the mean square displacement (MSD) was calculated for each nanoparticle over time (Δ*t* = 0, 4 s) following eq 1. Only particles within the 100 and 400 nm size range were analyzed, avoiding aggregates or incomplete nanoparticles. Finally, using an internally developed R script, the diffusion coefficients (*D*_0_ for nanomotors in the absence of fuel and *D*_eff_ for nanomotors in the presence of their respective analytes) were obtained from the linear region of the MSD vs Δ*t*, according to eq 2.

Spatial trajectories were also plotted using the same procedure.

1 $$\mathrm{MSD}={\{\left(x_{t}-x_{0}\right)\}}^{2}=1/N\overset{N}{\underset{i=0}{\sum }}{\left(x^{i}\left(t\right)-x^{t}\left(0\right)\right)}^{2}$$

where *N* is the number of nanoparticles, and *x_i_* and *x_t_* represent the position vectors of the particles at different time points.

2 $$\mathrm{MSD}=\left(4D_{\mathrm{eff}}\right)\cdot \Delta t,//,\mathrm{MSD}=\left(4D_{0}\right)\cdot \Delta t$$

### MTT Cell Viability Evaluation against J-Au/GOx-bor-(DOXO)-Ir

Cell viability was evaluated using the standard 3-(4,5-dimethylthiazol-2-yl)-2,5-diphenyl tetrazolium bromide (MTT) assay.^37^ Briefly, HeLa cells (10 000 cells per well) were seeded in 96-well plates in 160 μL of IMDM and allowed to adhere for 24 h. To synchronize endocytosis, cells were incubated at 4 °C for 10 min. The medium was then removed and replaced with nanomachine suspensions at concentrations of 25 and 50 μg mL^–1^, respectively, in Dulbecco’s phosphate-buffered saline (D-PBS, containing Ca^2+^ and Mg^2+^) supplemented with 1% antibiotics, followed by incubation at 37 °C for 30 min. Control wells received D-PBS containing 1% antibiotics only.

After two washes with D-PBS, cells were incubated for 24 h in culture medium supplemented with 5% FBS and varying concentrations of d-glucose (0, 25, 50, or 100 mM). Subsequently, 20 μL of MTT solution (5 mg mL^–1^ in PBS) was added to each well, and the plates were incubated for 3 h at 37 °C in a CO_2_ incubator. The medium was then replaced with 150 μL of dimethyl sulfoxide (DMSO) and incubated for 1 h to dissolve the formazan crystals. Absorbance was measured at 570 nm using a BioTek Instruments ELX800 plate reader.

### Confocal Fluorescence Microscopy

For confocal laser scanning microscopy, HeLa cells were seeded at a density of 60 000 cells per well in 24-well plates containing 12 mm diameter glass coverslips and incubated at 37 °C in a CO_2_ incubator. After 24 h, cells were treated with nanorobots following the same protocol used for cell viability assays and subsequently incubated in culture medium supplemented with 0 or 25 mM d-glucose.

After 4, 6, or 24 h of incubation, the medium was removed and cells were fixed at room temperature with 10% formalin in PBS, followed by permeabilization with 0.1% Triton X-100 for 5 min. Cells were washed three times with PBS and blocked with 2% bovine serum albumin (BSA) in PBS for 10 min. Cell membranes were stained with wheat germ agglutinin (WGA) conjugated to Alexa Fluor 488 (2 μg mL^–1^) for 10 min, followed by nuclear staining with Hoechst 33342 (1 μg mL^–1^) for 5 min. All staining steps were performed protected from light and followed by three washes with PBS.

Coverslips were mounted onto microscope slides and examined using an Olympus Life Science FluoView 1200 confocal laser scanning microscope equipped with a 60× oil-immersion objective. Images (1024 × 1024 pixels) were processed using ImageJ Fiji software.

## Supplementary Material



## Notes

Materials and methods: materials, general techniques, preparation of Janus gold mesoporous silica nanoparticles (J-Au), quantification of enzymatic activity for JAu/ GOx-bor-(Ru)-Ir and J-Au/AOX/esterase-bor-(Ru)-Ir nanomachines; enzyme inactivation for J-Au/GOx-bor-(Ru)-Ir and J-Au/AOX/esterase-bor-(Ru)-Ir nanomachines; cell culture; STEM image of the prepared IrNPs (Figure S1); characterization of MSN, J-Au and J-Au-bor-(Ru)-Ir using XRD analysis (A–B), characterization of MSN, J-Au, J-Au-bor, and J-Au-bor-(Ru)-Ir using thermogravimetric analysis (C), and FT-IR analysis (D) (Figure.S2); (A) nitrogen adosrption-desorption isotherms and (B) pore size distribution of MSN, J-Au, and J-Au-bor-(Ru)-Ir nanoparticles (Figure S3); kinetics of dye release from the nanomachine J-Au-bor-(Ru)-Ir in the absence and presence of HCl (Figure S4); relative dye release of active (red) and thermal inactivated (blue): (A) JAu/GOx-bor-(Ru)-Ir and (B) J-Au/AOX/esterase-bor-(Ru)-Ir, 2 h incubation time in 5 mM PB (pH 7.5) containing 100 mM of d-glucose for J-Au/GOx-bor-(Ru)-Ir or 10 mM ethyl acetate and 200 mM methanol for J-Au/AOX/esterase-bor-(Ru)-Ir, control experiments with J-Au/GOx-bor-(Ru)-Ir/J-Au/AOX/esterase-bor-(Ru)-Ir without substrates, respectively (Figure S5) (PDF)
