# Supplementary material for: Iridium Nanoparticle-Gated Janus Nanomachines for Enzyme-Controlled Drug Delivery
Source: ACS Appl Nano Mater. 2026 Aug 3;9(32):15147–60. doi: 10.1021/acsanm.6c01043 (PMC13488141; doi:10.1021/acsanm.6c01043)
Supplement: Supplementary file 1 [file an6c01043_si_001.pdf]

Supporting Information

Iridium Nanoparticle-Gated Janus Nanomachines for Enzyme-Controlled Drug Delivery

Beatriz Mayol[1,2]‡, Marta González-Jiménez[1]‡, Alfredo Sánchez[1], Susana Portela[1], Sandra Pradana-López[1,3,5], Carolina López-Benet[3], Teresa Moreno[1], RuiYuan Cai[1], Paloma Martínez-Ruiz[1], Narcisa Martínez-Quiles[4], Paula Díez[3,5,6], Ramón Martínez-Máñez[3,5,6,7], Irene Ojeda[1], Diana Vilela[1] and Reynaldo Villalonga[1]**

[1]Nanosensors and Nanomachines Group, Department of Analytical Chemistry, Faculty of Chemistry, Complutense University of Madrid, 28040 Madrid, Spain. E-mail: divilela@ucm.es, rvillalonga@quim.ucm.es.

[2]Department of Electrical and Computer Engineering, Rice University, Houston, TX, 77005, USA.

[3]Instituto Interuniversitario de Investigación de Reconocimiento Molecular y Desarrollo Tecnológico (IDM), Universitat Politècnica de València, Universitat de València, Camino de Vera, s/n., 46022 València, Spain.

[4]Department of Immunology, Ophthalmology and ENT, School of Medicine, Complutense University, 28040 Madrid, Spain.

[5]Unidad Mixta de Investigación en Nanomedicina y Sensores. Universitat Politècnica de València, Instituto de Investigación Sanitaria La Fe (IIS La Fe), Valencia, Spain.

[6]CIBER de Bioingeniería, Biomateriales y Nanomedicina, Instituto de Salud Carlos III, Bellaterra, 08913 Spain.

[7]Unidad Mixta UPV-CIPF de Investigación en Mecanismos de Enfermedades y Nanomedicina, Universitat Politècnica de València, Centro de Investigación Príncipe Felipe. Eduardo Primo Yúfera 3. 46012, València, Spain.

‡ These authors contributed equally to this work.

MATERIALS AND METHODS

1. Materials

Tetraethoxysilane (TEOS), 3-glycidoxypropyltrimethoxysilane (GPTMS), 3-mercaptopropionic acid, 3-aminophenylboronic acid, sodium borohydride ($NaBH_4$), trisodium citrate dihydrate, D-lactose, D-fructose, D-galactose, sucrose, 1-ethyl-3-(3-dimethylaminopropyl)carbodiimide (EDC), 2,2’-azino-bis(3-ethylbenzothiazoline-6-sulfonic acid) (ABTS), horseradish peroxidase (HRP), p-nitrophenyl acetate, iridium (III) chloride hydrated, glucose oxidase from Aspergillus niger (GOx), alcohol oxidase from *Pichia pastoris* (AOX) and esterase from Porcine liver were purchased from Sigma-Aldrich (USA). Hexadecyltrimethylammonium bromide (CTAB), tetrachloroauric acid trihydrate, 3-mercaptopropyltrimethoxysilane (MPTMS), N-hydroxysuccinimide (NHS), cystamine dihydrochlorided and D-glucose, were purchased from Alfa Aesar (USA).

Ethyl bromoacetate, ethyl butirate, Isocove´s modified Dubecco's medium (IMDM), fetal bovine serum (FBS), Wheat germ agglutinin (WGA) conjugated to Alexa Flour-488 and Hoechst 33342 were purchased from Thermo Fisher. Doxorubicin (DOXO) was acquired from Sequoia Research Products Ltd. (USA). Tris (2,2'-bipyridine)ruthenium (II) chloride hexahydrate was purchased from Acros organic (Belgium). Sodium hydroxide was purchased from Gerbu Biotechnik (Germany). Solvents were provided by Scharlau (Spain).

2. General techniques

Nitrogen adsorption/desorption isotherms and pore size distribution were determined using a physisorption analyzer (ASAP 2020, Micromeritics, USA). Infrared spectra were collected via Fourier-transform infrared spectroscopy using a Spectrum 400 Series instrument (Perkin Elmer, USA). The crystalline phases of the simples were examined by powder X-ray diffraction (XRD) using a X'Pert MRD diffractometer (PANalytical B.V., The Netherlands). Thermal properties were evaluated using a SDT-Q600 thermal analysis system (TA Instruments, USA). Transmission electron microscopy (TEM) images were obtained with a JEM-2100 microscope (JEOL LTd., Japan) and scanning transmission electron microscopy (STEM) images were recorded using JEM 3000F microscope (JEOL LTd., Japan). UV-visible absoption spectra were measured using an Ultroscope™ 8000 dual-beam spectrophotometer (Biochrom, UK). Dynamic light scattering (DLS) measurements were driven using Malvern Zetasizer instrument (Nanosizer ZS, Malvern Instruments, Ltd.-Malvern, United Kingdom). Confocal Fluorescence images were recorded using (Olympus Life Science). Cell viability % measurements were measured using a Biotek ELX800 plate reader.

3. Preparation of Janus gold mesoporous silica nanoparticles (J-Au)

First, MSNPs were synthesized following a previously reported procedure with minor modifications.[1] Briefly, 1.0 g of cetyltrimethylammonium bromide (CTAB) was dissolved in 0.48 L of distilled water in a 1 L three-necked round-bottom flask. Subsequently, 3.5 mL of a 2.0 mol L^{-1} NaOH aqueous solution were added. The mixture was equipped with a reflux condenser and heated in a silicone oil bath at 80 °C. Once the temperature was stabilized, 5.0 mL of tetraethyl orthosilicate (TEOS) was added dropwise under vigorous stirring. After completion of the reaction, the mixture was cooled to room temperature, and the resulting white precipitate was recovered by filtration, washed

thoroughly with distilled water and methanol, and dried at 60 °C for 18 h. The obtained solid was finely ground and calcined at 550 °C for 5 h to remove the CTAB, the structure-directing agent.

In parallel, gold nanoparticles (AuNPs) were synthesized according to a previously reported citrate reduction method.[2] Briefly, 100 mL of an aqueous $HAuCl_4$ solution (0.3 mM) was heated to boiling without stirring. Upon reaching reflux, 5 mL of trisodium citrate solution (39 mM) was rapidly added. The mixture was then stirred and maintained at boiling for 10 min, during which a characteristic color change from pale yellow to deep wine red was observed, indicating nanoparticle formation. The suspension was allowed to cool to room temperature, and the procedure was repeated until a total volume of 500 mL of AuNP dispersion was obtained.

Then, Au-MSN Janus nanoparticles were prepared following a previously reported method developed in our group.[2] Typically, 200 mg of MSNPs were dispersed in 10 mL of an aqueous solution containing CTAB (1 μmol L^{-1}) and ethanol (6.7% v/v). The suspension was heated to 75 °C in a water bath, after which 1.0 g of paraffin wax was added. Once the wax had completely melted, the mixture was emulsified using an Ultra-Turrax T-10 homogenizer (IKA, Germany) at 25000 rpm for 10 min while maintaining the temperature at 75 °C. The resulting emulsion was subsequently magnetically stirred for 1 h at the same temperature, cooled to room temperature, and diluted with 10 mL of methanol. To selectively introduce thiol groups onto the exposed silica surface, 200 μL of (3-mercaptopropyl)trimethoxysilane (MPTS) were added, and the reaction mixture was stirred for 3 h at room temperature. The resulting solid was collected by filtration using a 47 mm diameter, 0.22 μm nylon membrane and thoroughly washed with methanol.

Finally, the thiolated MSNPs were dispersed in 500 mL of freshly prepared AuNP solution and stirred overnight at room temperature to promote gold attachment. The product was isolated by filtration using a 47 mm, 0.22 μm nylon membrane and washed sequentially with methanol and methanol/chloroform mixtures of increasing chloroform content to ensure complete removal of paraffin wax. After drying at 60 °C, the resulting Janus-Au MSNPs (J-Au) were stored until further use.

4. Quantification of enzymatic activity for J-Au/GOx-bor-(Ru)-Ir and J-Au/AOX/Esterase-bor-(Ru)-Ir nanomachines.

Glucose oxidase (GOx) activity was determined by a colorimetric assay using horseradish peroxidase (HRP) as a coupled enzyme.[3] The assay is based on the HRP-mediated oxidation of 2-2'-azino-bis(3-ehtylbenzothiazoline-6-sulfonic acid) diammonium salt (ABTS) in the presence of H_2O_2 generated during GOx-mediated oxidation of D-glucose to gluconic acid. The increase in the absorbance was monitored at 405 nm, corresponding to the oxidation of ABTS (ABTSox). Enzymatic activity was calculated using the molar extinction coefficient of oxidized ABTS (ε_{ABTSox} = 36.8 $mM^{-1}\cdot cm^{-1}$).

In a similar manner, alcohol oxidase (AOX) catalyzes the oxidation of alcohols to the corresponding aldehydes generating H_2O_2 as a byproduct. In this assay, methanol was used as the substrate to induce H_2O_2 production. The enzymatic activity was determined by a colorimetric method in the presence of horseradish peroxidase (HRP) in which H_2O_2 reacts with ABTS and the increase in the absorbance is monitored at 405 nm. Enzymatic activity was calculated using the molar extinction coefficient of oxidized ABTS (ε_{ABTSox} = 36.8 $mM^{-1}\cdot cm^{-1}$).

Esterase exhibits hydrolase activity and catalyzes the hydrolysis of ester groups into the corresponding alcohols and acids. Esterase activity was determined by a colorimetric assay using p-nitrophenyl acetate as the substrate.[4] The reaction kinetics were monitored spectrophotometrically at 415 nm. Enzymatic activity was calculated using the molar extinction coefficient of p-nitrophenol ($\varepsilon_{p\text{-nitrophenol}}$ = 13.9 $mM^{-1}\cdot cm^{-1}$).

5. Enzyme inactivation for J-Au/GOx-bor-(Ru)-Ir and J-Au/AOX/esterase-bor-(Ru)-Ir nanomachines.

Enzyme inactivation was achieved by thermal treatment by immersing aqueous suspensions of J-Au/GOx-bor-(Ru)-Ir or J-Au/AOX/Esterase-bor-(Ru)-Ir nanomachines in a water bath maintained at 100 °C for 30 min. After cooling to room temperature, release experiments were conducted following a protocol analogous to that used for standard release assays. Briefly, the thermally treated nanomachines were incubated with the corresponding substrates, D-glucose (100 mM) for J-Au/GOx-bor-(Ru)-Ir or ethyl acetate (10 mM) and methanol (200 mM) for J-Au/AOX/Esterase-bor-(Ru)-Ir, and the absorbance of the supernatant was recorded at 454 nm after 120 min of incubation to confirm the loss of enzymatic activity.

6. Cell culture

HeLa human cervical adenocarcinoma cells (ATCC) were cultured in Iscove's Modified Dulbecco's Medium (IMDM) containing 25 mM D-glucose and supplemented with 10%

fetal bovine serum (FBS) and antibiotics (penicillin, 100 U mL^{-1}; streptomycin, 100 μg mL^{-1}). Cells were maintained at 37 °C in a humidified atmosphere containing 5% CO_2 and subcultured two to three times per week by trypsinization.

List of Figures:

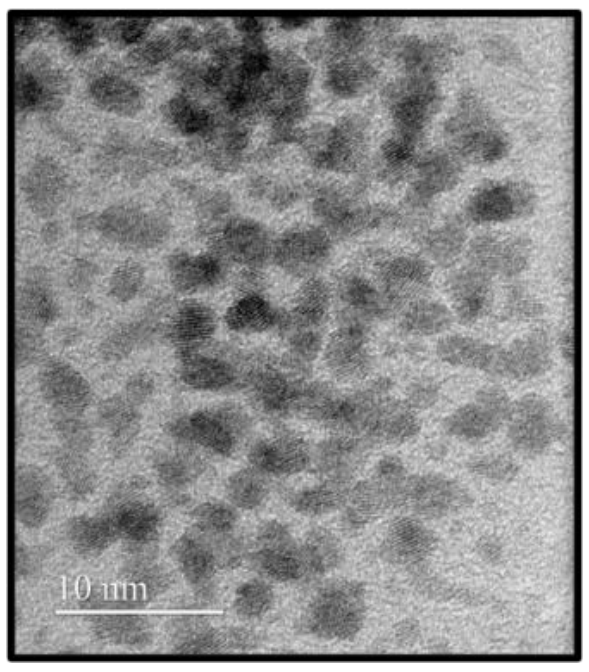

Fig S1: STEM image of the prepared IrNPs.

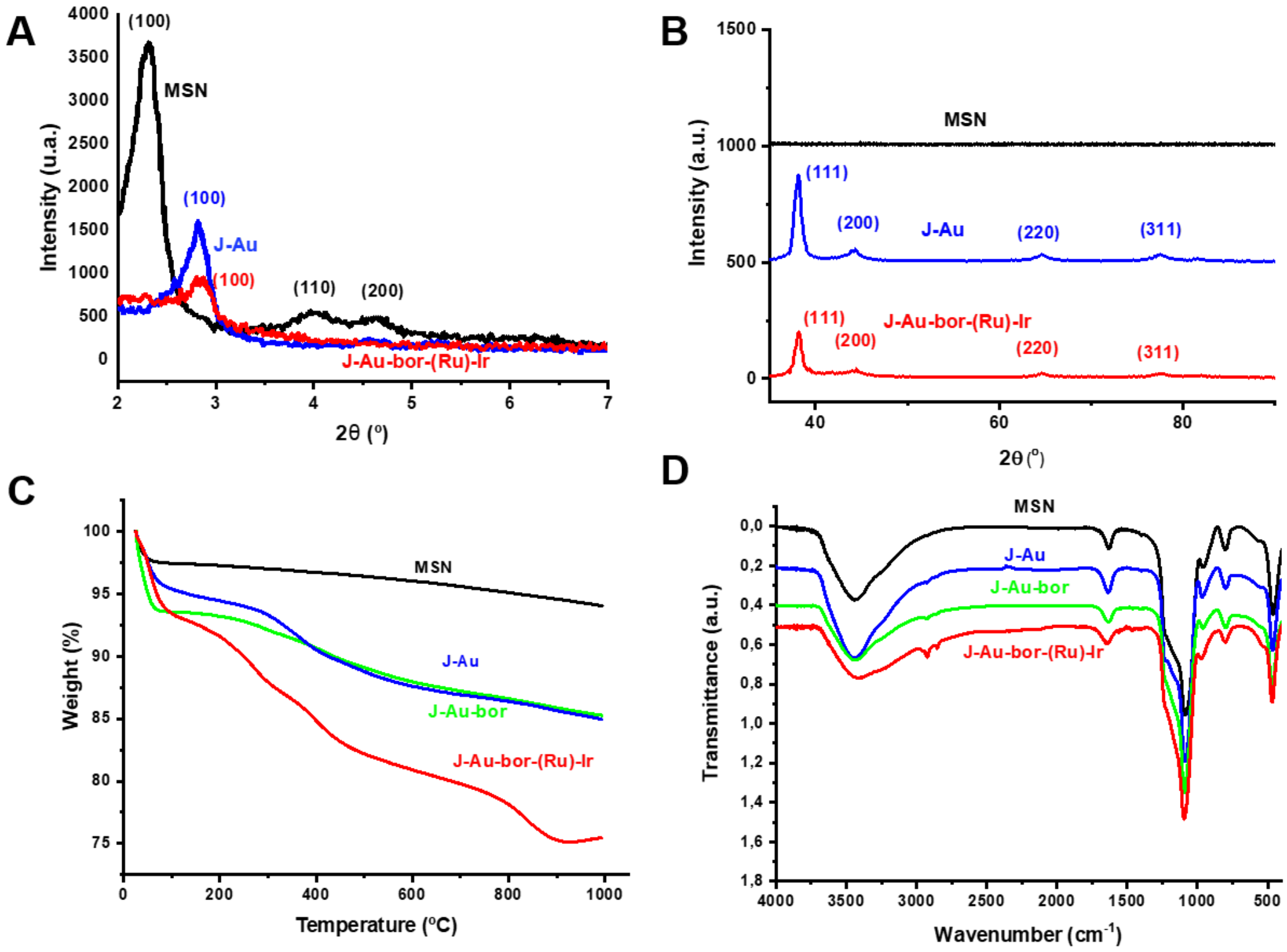

Fig S2: Characterization of MSN, J-Au and J-Au-bor-(Ru)-Ir using XRD analysis (**A-B**). Characterization of MSN, J-Au, J-Au-bor and J-Au-bor-(Ru)-Ir using thermogravimetric analysis (**C**) and FT-IR analysis (**D**).

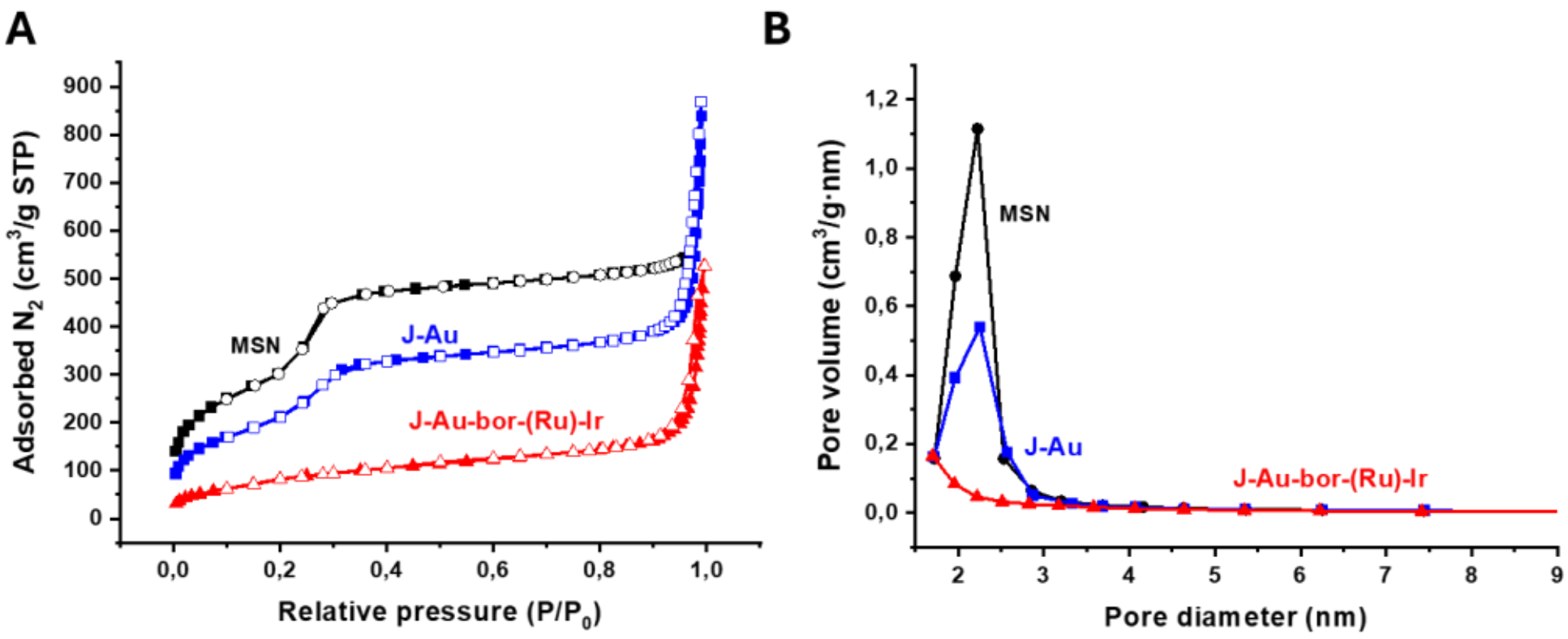

Fig S3: (**A**) Nitrogen adsorption-desorption isotherms and (**B**) pore size distribution of MSN, J-Au y J-Au -bor-(Ru)-Ir nanoparticles.

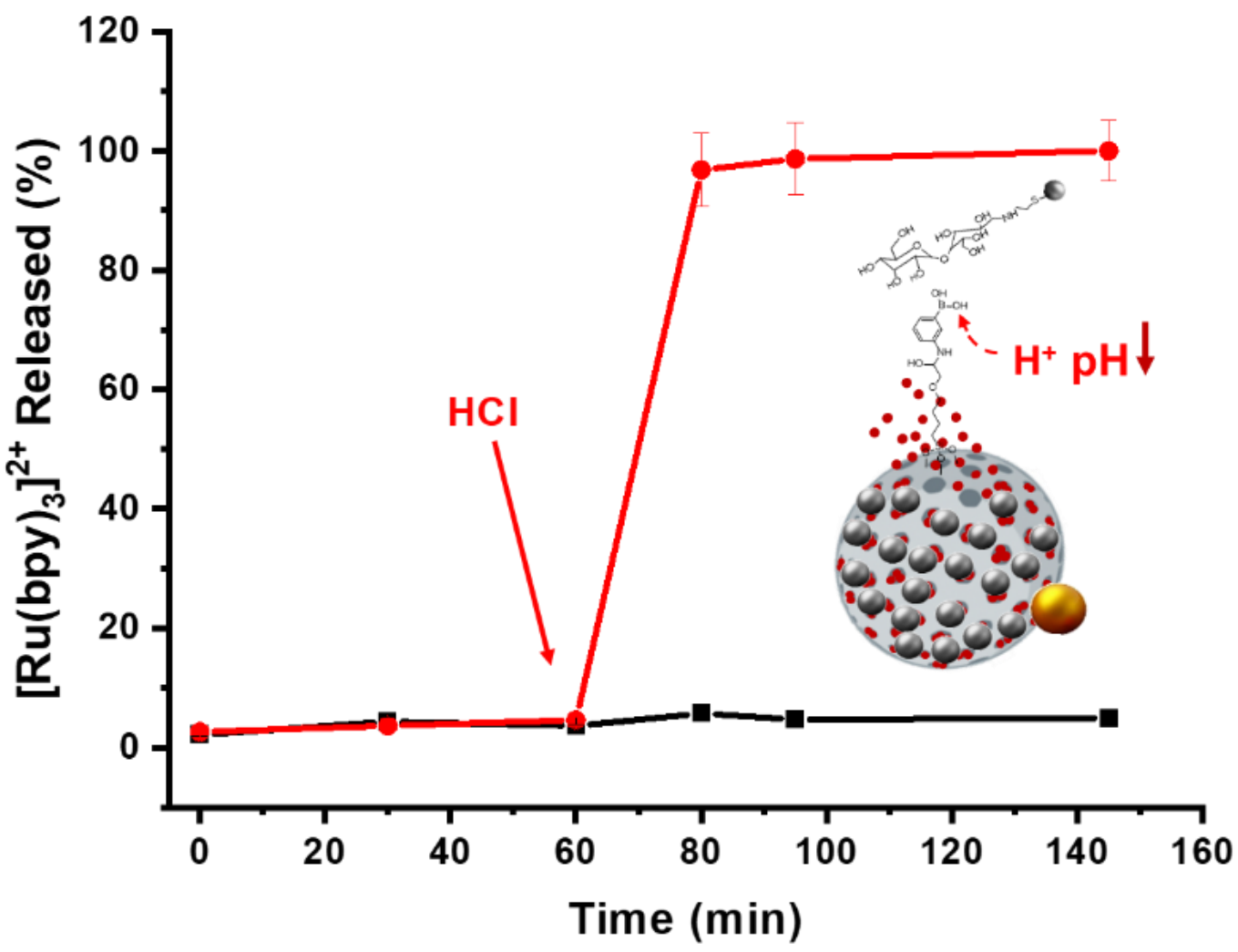

Fig S4: Kinetics of dye release from the nanomachine J-Au-bor-(Ru)-Ir in the absence and presence of HCl.

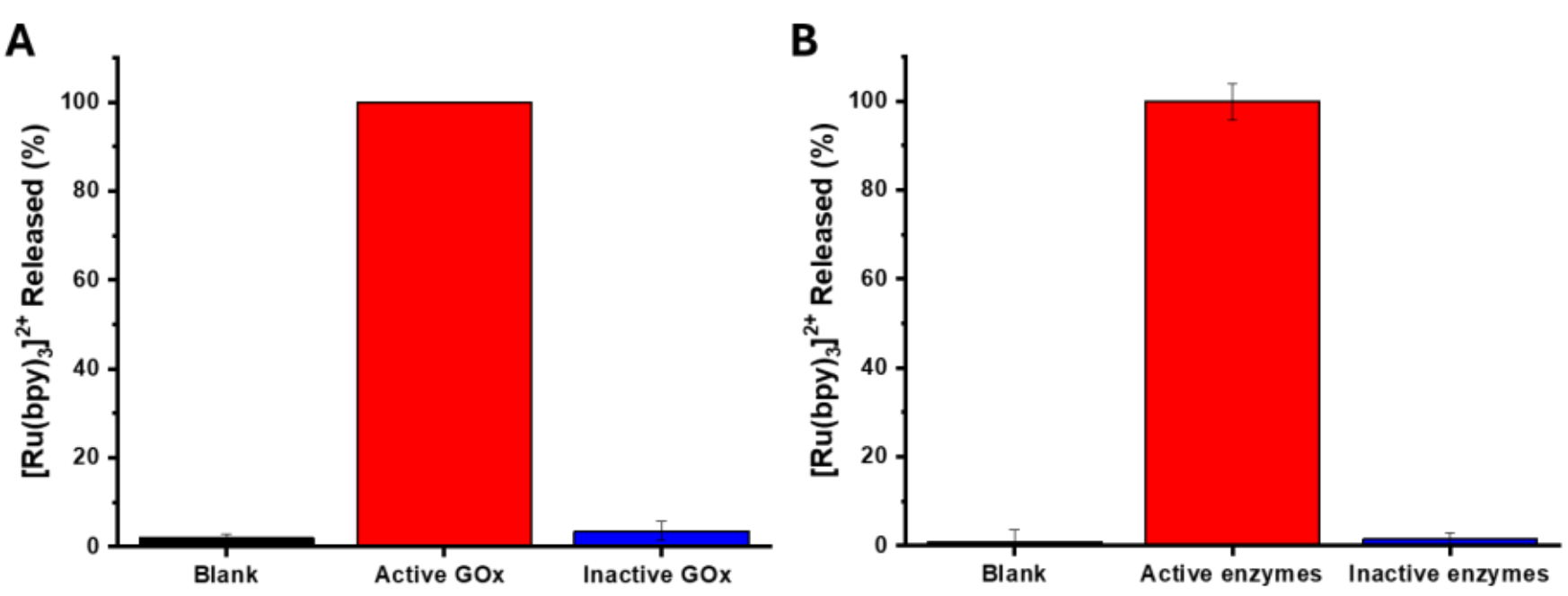

Fig S5: Relative dye release of active (red) and thermal inactivated (blue) (A) J-Au/GOx-bor-(Ru)-Ir and (B) J-Au/AOX/esterase-bor-(Ru)-Ir. 2 h incubation time in 5 mM PB (pH 7.5) containing 100 mM of D-glucose for J-Au/GOx-bor-(Ru)-Ir or 10 mM ethyl acetate and 200 mM methanol for J-Au/AOX/esterase-bor-(Ru)-Ir. Control experiments (black) with active J-Au/GOx-bor-(Ru)-Ir / J-Au/AOX/Esterase-bor-(Ru)-Ir without substrates, respectively.

References

(1) Villalonga, R.; Díez, P.; Sánchez, A.; Aznar, E.; Martínez-Máñez, R.; Pingarrón, J. M. Enzyme-Controlled Sensing–Actuating Nanomachine Based on Janus Au–Mesoporous Silica Nanoparticles. *Chemistry A European J* **2013**, *19* (24), 7889–7894. https://doi.org/10.1002/chem.201300723.
(2) FRENS, G. Controlled Nucleation for the Regulation of the Particle Size in Monodisperse Gold Suspensions. *Nature Physical Science* **1973**, *241* (105), 20–22. https://doi.org/10.1038/physci241020a0.
(3) Bateman, R. C.; Evans, J. A. Using the Glucose Oxidase/Peroxidase System in Enzyme Kinetics. *J. Chem. Educ.* **1995**, *72* (12), A240. https://doi.org/10.1021/ed072pA240.
(4) Fredrikzon, B.; Olivecrona, T. Decrease of Lipase and Esterase Activities in Intestinal Contents of Newborn Infants during Test Meals. *Pediatr Res* **1978**, *12* (5), 631–634. https://doi.org/10.1203/00006450-197805000-00004.
